# Structured fragment-based object tracking using discrimination, uniqueness, and validity selection

**DOI:** 10.1007/s00530-017-0556-7

**Published:** 2017-06-29

**Authors:** Jin Zheng, Bo Li, Ming Xin, Gang Luo

**Affiliations:** 1 Beijing Key Laboratory of Digital Media, School of Computer Science and Engineering, Beihang University, Beijing 100191, China; 2 Schepens Eye Research Institute, Mass Eye and Ear, Harvard Medical School, Boston, MA 02114, USA

**Keywords:** Fragment-based tracking, Structured fragment, Discriminative and unique feature, Harris-SIFT filter, Template update

## Abstract

Local features have widely been used in visual tracking to improve robustness in the presence of partial occlusion, deformation, and rotation. In this paper, a local fragment-based object tracking algorithm is proposed. Unlike many existing fragment-based algorithms using all the fragments and allocating the weight to each fragment according to similarity, the proposed algorithm only selects discriminative, unique, and valid fragments for tracking. First, discrimination and uniqueness metric are defined for each local fragment, and an automatic pre-selection mechanism is proposed for all these fragments. Second, a Harris-SIFT filter is used to select the current valid fragments and exclude the occluded or highly deformed fragments. By selecting the discriminative, unique, and valid fragments, these fragments are used to construct a structured description for the object. Finally, the object tracking is performed using the selected fragments combining the displacement and similarity, as well as spatial constraint of the selected fragments. The object template can be updated by fusing feature similarity and structural consistency. The experimental results on a recent OTB 2013 tracking benchmark data set demonstrate that the proposed algorithm can achieve reliable tracking results even in the presence of significant appearance changes, partial occlusion, and similar disturbances.

## 1 Introduction

Object tracking is one of the prerequisites for analyzing and understanding video content, which aims at automatically identifying the same object in consecutive frames from a video sequence once initialized. Due to dynamic environments, such as partial occlusion, various deformations, disturbances from similar object, illumination variation, and cluttered background, the accuracy of object tracking is largely degraded and far from the requirement in practical applications. Therefore, object tracking is a challenging task and lots of studies focus on this area [1–5].

Object tracking has been mostly formulated in a match-and-search framework. In this framework, there are two important steps in the tracking process, i.e., appearance modeling and motion searching. It is critical to handle appearance changes of objects in dynamic and cluttered environments, where the tracking performance will be robust. Therefore, appearance models including the most salient appearance features contained in the object itself, and the valid appearance features which can handle appearance changes in the presence of various disturbances, are of significant importance for object tracking techniques.

In general, visual trackers can be categorized into two groups based on appearance models, i.e., generative models [6–8] and discriminative models [9–14]. A generative tracker generally employs a learnt appearance model to identify the candidate with the maximal similarity to the appearance model, whereas a discriminative tracker formulates the tracking problem as a binary classification task to identify the decision boundary for separating the target object from the background. In the recent literature, many sophisticated and advanced learning trackers have been proposed [15, 16]. Some representative learning-based methods in visual tracking include: boosting [12], multiple instance learning [17], structured output SVM [18], and deep learning [19–21]. Recent benchmark analysis has shown that the top performing tracking algorithms are mostly discriminative trackers [22] or hybrid trackers [23].

**Fig. 1:**
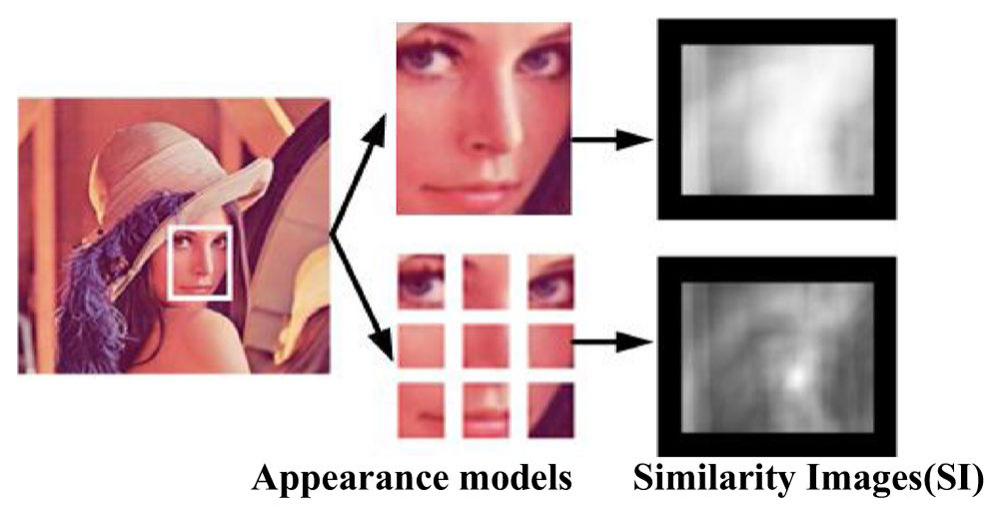
Similarity images of conventional color histogram vs. fragment-based color histogram

Meanwhile, it should be noted that not all parts of the object can be distinguished from the background. In other words, not all the regional parts can be considered as discriminative. Therefore, discrimination is often described by associating with the local object regions. Fragment-based object representation can desirably illustrate this local representation. Although the object can be represented by an indivisible whole, fragment-based representation can adopt multiple image fragments, which are multiple rectangles covering the object region to describe the object. Accordingly, the tracking result is obtained using these multiple fragments. An illustrative example is shown in Fig. 1. The color histogram is used to describe an object, and two-color appearance models are adopted for the Lena face. The top row presents the similarity matching results using the conventional color histogram obtained from the whole Lena face. In contrast, the results using fragment-based color histogram are given in the bottom row which divides the face image uniformly to get nine rectangular fragments and the average similarity values of all the fragments are the similarity of the corresponding object. The location of an object with higher matching similarity is assigned a brighter value in similarity images. Apparently, color histogram of the whole object fails to differentiate the salience among different locations of the face, since the brightness of the face region (the correct location) and the hat region (the incorrect location) are similar. This is the reason that the accuracy of similarity is degenerated. However, fragment-based color histogram is capable of distinguishing the difference and accurately locating the object. More concretely, the brightest spot in similarity image is also the center of the object.

**Fig. 2:**
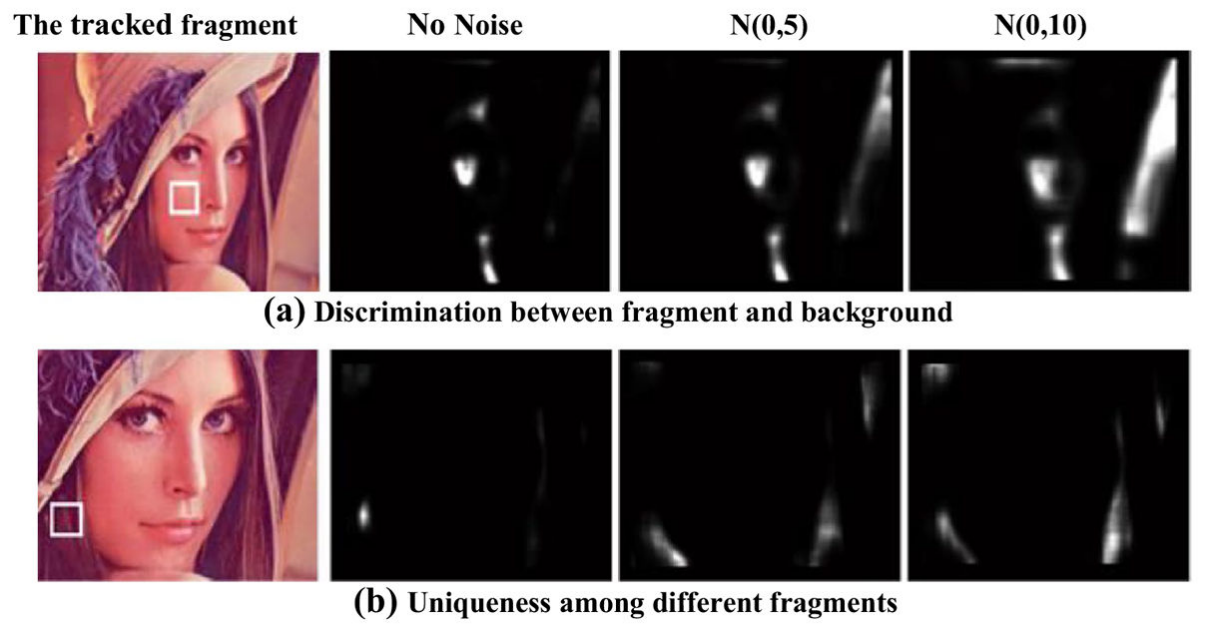
Discrimination and uniqueness illustration

Although fragment-based representation is effective, it may not be optimum with equally treated fragments. The existing methods often consider the discrimination between object fragment and its neighboring background, which is reasonable and can be illustrated in Fig. 2a. The fragment with a white square in Lena face has similar color as the background. When noise exists, for example a Gaussian distributed noise of *N*(0,10), the similarity to the background is even higher than the similarity to the true fragment. Therefore, it is more appropriate to use the fragments which are distinctive from background. In addition, it should be noted that uniqueness is another critical issue. More specifically, the fragments should not only differ from the background, but also differ from other fragments. Let us consider the region of Lena’s hair as an example. With increased noises, the hair block marked by a white square becomes similar to other regions containing hair, as shown in Fig. 2b. In other words, the marked hair fragment cannot be considered unique. If it is occluded, the tracker will mistakenly locate the other hair fragments with a high probability. Therefore, the uniqueness of selected fragment should also be considered for accurate tracking.

**Fig. 3:**
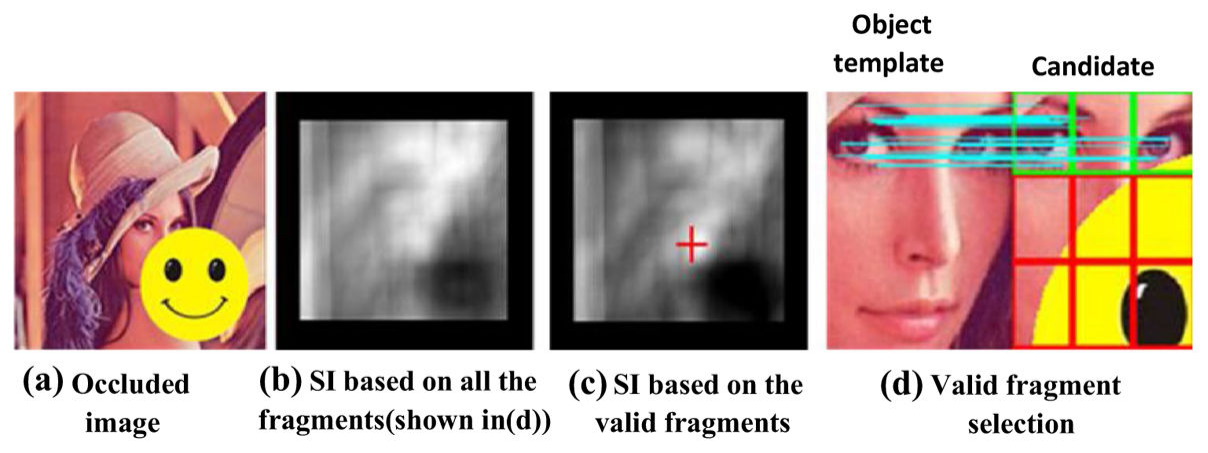
Fragment-based tracking in occlusion situation

For many tracking applications, occlusion frequently occurs. If occluded portions are directly used in tracking, it is highly possible that the tracking is biased, as illustrated in Fig. 3b. If unoccluded fragments are used in tracking as shown in the green rectangles in Fig. 3d, the object can be correctly located with only one-third of the fragments, as shown in the red cross in Fig. 3c. The other fragments can be considered as invalid fragments due to occlusion or lack of discriminative features. The optimal rules of selecting valid fragments or assigning weights to fragments are fundamental problems in fragment-based methods. As an example shown in Fig. 3b, the correct Lena’s face and incorrect locations such as the hat region both have higher color histogram similarities. Therefore, color histogram similarity can be appropriately used for con-firming the candidates, but cannot locate the unique correct location accurately, especially in occlusion, similar background, and noise disturbance situations. At the same time, the localized accuracy of color histogram similarity is somewhat low. Using histogram calculation to validate the fragments and then using those validated fragments with histogram similarity weight to track is analogous to circular reasoning. This is the essential limitation for the existing weighted fragment-based tracking methods using only color histogram.

Notably, the discrimination and uniqueness can reflect the intrinsic salient characteristic of the object as well as its validity, which can reflect the local valid parts at the current time. In this paper, an object tracking algorithm is proposed using and combining multiple validation measures to prevent erroneous tracking. For the first validation, a discriminative and unique measure is used for evaluating the local fragments. This measure can describe the discriminative difference between the fragment and background, and the unique difference between a particular fragment and the other fragments for the tracked object. Therefore, the proposed algorithm determines discriminative and unique fragments (DUFrags), which will eliminate the confusing fragments and reduce the costs of fragment search. For further validation, validated DUFrags (V_DUFrags) will be selected by Harris-SIFT filter. The filtering process is performed with each frame, and the filtered results can eliminate the object parts which are disturbed at the current frame. In particular, the Harris-SIFT is insensitive to scale changes, rotation, and illumination changes, where tracking can be performed in a more robust manner. Moreover, the local characteristic of Harris-SIFT also makes the overall object tracking robust to partial occlusion. An important thing to be noted here is that Harris-SIFT allows us to use the highly localized features for tracking. Furthermore, the proposed algorithm allows V_DUFrags to move away from each other with certain spatial constraints for locating the object of interest. This strategy will improve robustness of the algorithm to subtle local changes in object appearance. Meanwhile, V_DUFrags with geometric structures are capable of recognizing the geometric deformable object, such as rotation and scale changes. Therefore, V_DUFrags-based representation is more efficient compared with pixel-based representation. It can be concluded that with the validation procedure based on Harris-SIFT filtering and spatial structure constraints, the flexible fragment grid will not necessarily result in object drifting.

Hence, the proposed fragment-based tracking algorithm can utilize object features at three levels, (a) global level—a group of structured fragments to describe the object to be tracked; (b) a medium level—each V_DUFrag is tracked based on its regional features; (c) a low level—Harris corners within each V_DUFrag can be used for accurate localization. With the aforementioned multiple validation measures and these three levels of features, the proposed algorithm is capable of tracking the object even under significant appearance changes, partial occlusion, and similar disturbances.

The rest of this paper is organized as follows. Section 2 introduces the related works. Section 3 describes the proposed algorithm. Experimental results and discussions are presented in Sect. 4. Section 5 gives the concluding remarks.

## 2 Related works

### 2.1 Recent object-representative developments

Over the last few decades, visual tracking has experienced rapid developments. A detailed and thorough summary of the recent advances in visual tracking is reviewed in [4, 5].

For two tracking processing steps, i.e., appearance modeling and motion searching, appearance modeling is particularly significant. The feature extractor plays a very important role for appearance modeling, and the features of appearance modeling can be categorized into global features and local features. For global features, they are sensitive to the changes in object itself and the surrounding background due to the lack of spatial constraints. To solve this problem, weighted histograms [24, 25] have been developed, which is constructed by calculating the distance between each pixel and the object center. This method will enhance the influence of the object and weaken the influence of the background. In this way, the partial occlusions on the peripheral regions of the object can be properly dealt with. However, it is still invalid when the occlusion moves closer to the center of the object. Moreover, it cannot distinguish all the different objects with the same color but with different color layouts. In addition, spatial histograms [26, 27] have also been proposed, which add the mean vector and covariance matrices of the spatial coordinates of the pixels contributing to each bin of the color histogram. These methods, however, can still become ineffective when the object has large shape change or occlusion.

As a matter of fact, occlusion or appearance changes occur in the objects to be tracked, global features will also undergo great variations, and oftentimes, they are hard to determine where the appearance changes happen and which part is still available. On the contrary, local features can provide spatial information, which are more robust in the presence of object deformation and partial occlusion. Therefore, local features have been widely used. For example, Appearance Learning In Evidential Nuisance (ALIEN) [28] proposes a novel object representation based on the weakly aligned multi-instance local features, where local features are used for detecting and tracking. Flock of Trackers (FoT) [29] estimates the object motion using local trackers covering the object. Matrioska [30] also proposes to use local features and a voting procedure, where the detection module uses multiple key point-based methods (ORB, FREAK, BRISK, SURF, etc.) inside a fallback model to localize the object correctly. Similarly, Kwang [31] describes the object using feature points, and utilizes motion saliency and description discrimination to select local features for tracking. Yang [32] proposes an attention visual tracking algorithm, which selects the discriminative regions based on minimum cross-entropy to locate the object. Goferman [33] proposes a context-aware saliency detection (CASD) method, which is based on four principles observed in the psychological literature. Although local feature-based methods are widely used, most of the existing methods only focus on discrimination and saliency. However, the different samples with similar discrimination degrees, or the similar samples existing in the different parts of the object, cannot be desirably differentiated by the tracker.

Apart from the appearance model used in a tracking system, the employed feature descriptor also plays a significant role in the final tracking performance. The HOG feature descriptor [34] is a popular feature descriptor used in visual tracking tasks. Various state-of-the-art object tracking algorithms, such as Struck [18], KCF [22], SDRCF [35], and Staple [36], employ HOG as the feature descriptor. However, HOG is primarily used to describe the edges of an object, which is not robust to the rotation and deformation of objects. In contrast, the color information can be considered to be stable when the object undergoes deformation. However, since the color information in the surrounding background of the object is often undesirable, the conventional color histogram lacks the spatial information. Therefore, straightforward usage of the color information in a tracking algorithm will ultimately lead to inaccurate training and detection.

### 2.2 Fragment-based tracking

Among these local feature-based methods, the most popular one is the patch-based or fragment-based tracking [37–60], such as Matrioska [30], FragTrack [37], LGT [55], DPT [60], etc. In these methods, the object is divided into several patches or fragments for tracking. For example, Adam et al. [37] divided the object region into several fragments and located the target’s position by fusing the voting maps of these fragments. Nejhum et al. [38] modeled the foreground object shape in terms of a small number of rectangular blocks. The algorithm can track the objects by matching foreground intensity histograms and updating the part-based appearance model on-the-fly. Kwon et al. [39] represented an object by a fixed number of local patches and updated the model during tracking. In contrast, Felzenszwalb et al. [48] developed a multi-scale deformable part model to detect and localize objects of a generic category. The part model is composed of a coarse global template for the entire object and high-resolution part templates. Liu et al. [49] exploited multiple patch-based correlation filters to obtain several response maps, where an adaptive weighting method is proposed to fuse these response maps for object tracking. Compared with the “holistic representation”-based trackers, i.e., use a single regular bounding box to describe the target, the fragment-based algorithms are more discriminative in distinguishing the object to be tracked from its surrounding background [50].

In summary, the existing patch-based or fragment-based tracking algorithms basically obtain features from color histogram [37, 40, 42, 45–47, 54], edge histogram [41], gradient histogram [44, 50, 53], harr-like features [59], SIFT features [52], motion feature [29], patch properties [51, 56], and the combination of multiple visual properties such as color, shape, and apparent local motion [55]. The partitions of fragment are either overlapping [37, 40–42, 45, 47] or non-overlapping [29, 43, 44, 46, 49–56]. In general, color and gradient are the most useful features, where the effects of overlapping or non-overlapping partition do not play a major role.

For most of the earlier reported fragment-based algorithms [37–47], each fragment is independently tracked based on features matching, and the whole object is tracked using linear weighting scheme, vote map, or maximum similarity of the fragment location. For most of the recently reported algorithms [48–59, 58], in contrast, spatial constraints between these fragments are often imposed, and these algorithms are more robust to deformation and illumination changes. Among these algorithms, graph representation-based method is a particularly popular method, which include inter-ARG and intra-ARG [51], attributed relational feature graph (ARFG) [52], dynamic graph(DG) [56], memory graph [57], star model [48], etc. Although graph representation is efficient compared with pixel-based representation, since it only requires a small number of features to model an object, these methods rely on the reliability of local features. The local features can be any distinctive features, such as SIFT features [61]. If the features are unreliable, the performance of the tracker will be degraded. In addition, once the object is represented as a graph, tracking is formulated as a graph matching problem. However, the abundant trivial fragmental parts also introduce problems for graph matching, and the complicated structure slows down the tracking speed. Therefore, the existing methods can only encode dependence between parts and the object center such as start model [48] or the relations of adjacent parts [51, 56], but the inter-part dependencies cannot be encoded.

By further analysis, graph matching involved similar local features as well as similar spatial layout, which means a cluster of spatially coherent correspondences between two sets of local features within two images, respectively [61–65]. For these methods, the main idea is to identify pairwises based on local features first, and then build the transformation matrix or compute a quadratic function based on the spatial constraints. These methods can be exactly considered in determining the valid object fragments in tracking. However, there are several issues that require special attention [61–65]: (1) there exists large amount of redundancy in pairwise distances among local features; (2) there may be large deformation; (3) different scales and orientations; (4) significant occlusions and outliers. Thus, the existing approaches are dedicated to address these issues in common visual pattern discovery. However, for tracking, the efficiency needs to be further improved. Frankly, our method for selecting the valid fragments is motivated by these above-mentioned methods combining feature similarity and space constraint, but spatial constraint is simplified considering the deformation and computational efficiency.

It should be noted, for improving the efficiency, that many fragment-based tracking algorithms consider that the position of each fragment with respect to the center of the object is rigid or even fixed [37, 41–47], or their relative positions among the patches are rigid [51]. This assumption inevitably reduces the flexibility against deformation. Moreover, for most of the algorithms, each fragment is equally treated and a different weight is assigned only based on its contribution [37, 40–47, 51, 53, 56]. To estimate the contribution of each fragment, the background is used, but the differences among the object fragments are ignored [41, 43, 45]. As a matter of fact, similar object fragments may cause confusion and ultimately lead to incorrect tracking result. Furthermore, some algorithms can locate the object only using one maximal similarity fragment [37, 41]. However, an individual fragment may have a larger chance of drifting due to the unreliable similarity scores, which is particularly severe in the presence of a cluttered background. Although some algorithms employ a linear-weighted location scheme of multi-fragments [40, 42–47], their performance is degraded as the number of unreliable fragments increases in complex environments. In these situations, the similarity scores of incorrectly tracked fragments become unreliable, where the use of many such fragments would affect tracking performance in a negative way. Therefore, it is proposed to incorporate fragment selection into tracking [29, 52, 54, 55]. The challenge becomes how to appropriately select the most salient characteristic rooted in the tracked object itself as well as the valid object characteristic, even in the complicated and diversified environment, which is also the motivation and focus of this paper.

## 3 The proposed algorithm

**Fig. 4:**
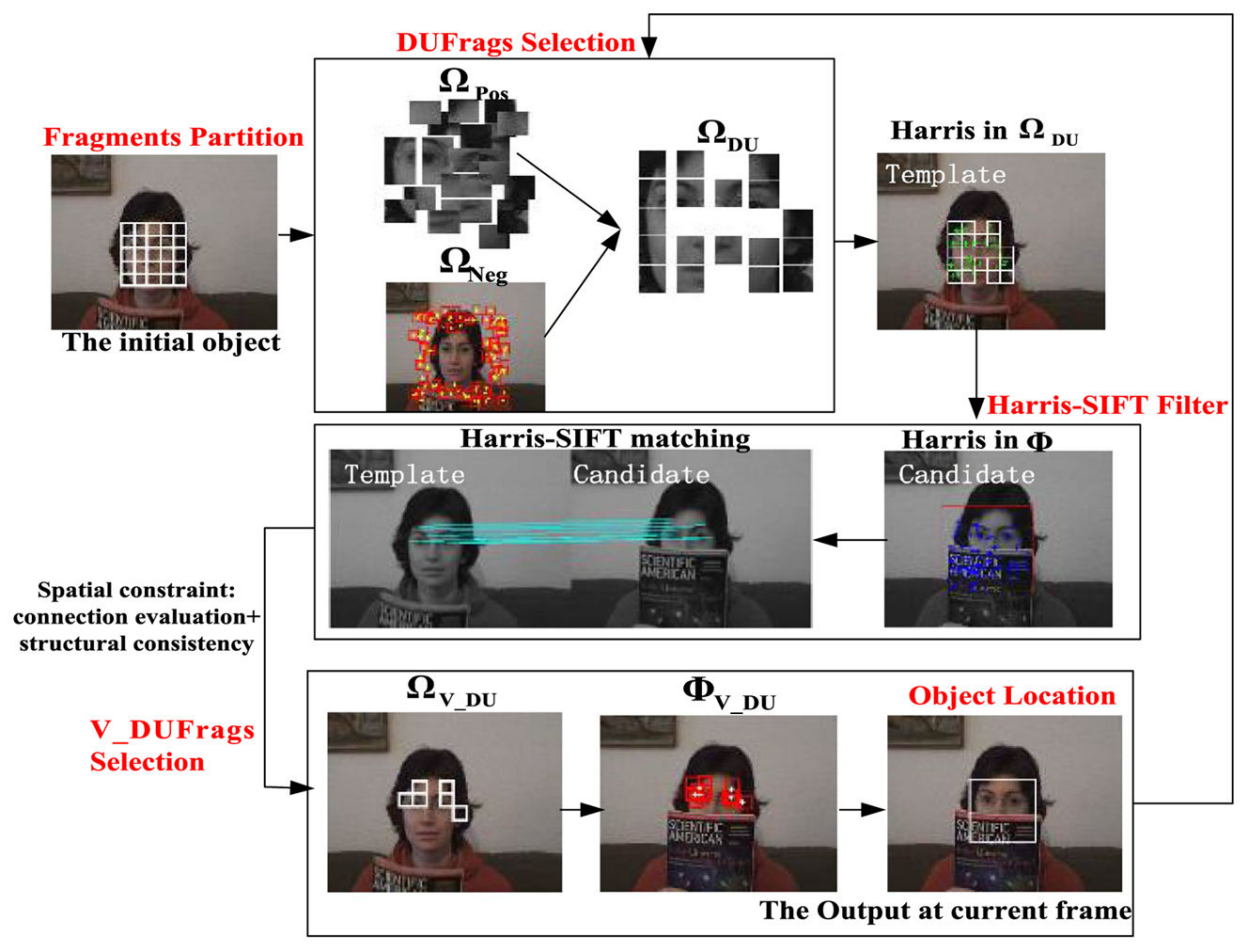
Framework of the proposed algorithm

Based on the above analysis, in this paper, a fragment-based tracking algorithm is proposed by selection of fragments. Assuming the appearance of an object is explicit and can be expressed in the detection phase, the discrimination and uniqueness are evaluated, and most discriminative and unique fragments (DUFrags) are selected. In the tracking phase, the Harris-SIFT filter is used which has desirable localization properties, to exclude the current invalid fragments corresponding to occlusion or too large appearance change. At the same time, connection evaluation and structural consistency are also used to validate the valid fragments. Subsequently, these Valid DUFrags (V_DUFrags) are used for motion searching. A joint likelihood can be obtained by combining fragment displacement and its brief, where the object of interest can be localized. The framework of the algorithm is illustrated in Fig. 4. All the abbreviations and symbols used throughout the paper are summarized in Table 1.

### 3.1 DUFrags selection based on discrimination and uniqueness

Given an object of interest, we first divide the bounding box of the object template into several rectangular fragments. The proposed algorithm adopts adjacent non-overlapping partition. The number of fragments depends on the size of the object. Since the object’s similarity is represented by color histogram features, the fragment should be large enough to provide a reliable histogram. On the other hand, the number of fragments should be large enough to contain sufficient spatial information. Based on the experiments, an object is empirically divided into squares of approximately 20-pixel wide. Moreover, HSV color histogram with 85 bins [66] is adopted, and Bhattacharyya distance is used to calculate the color histogram similarity.

Instead of using all the weighted fragments for tracking, a fragment pre-selection mechanism based on discrimination and uniqueness is performed. Assume that the object template **T** is divided into *N* fragments, which is represented as positive samples set $\Omega_{\mathrm{pos}}=\{F_{+1}^{t},F_{+2}^{t},\ldots ,F_{+N}^{t}\}$, and the negative samples set $\Omega_{\mathrm{Neg}}=\{F_{-1}^{t},F_{-2}^{t},\ldots ,F_{-M}^{t}\}$ existing in the neighboring background. The number of positive samples is denoted by *N*, the number of negative samples is denoted by *M*, and the time is denoted by *t*. The size of negative fragment is the same as positive fragment.

Discrimination is defined as $D_{i}^{t}$, which represents the discrimination degree between $F_{+i}^{t}$ and its background Ω***_Neg_***. Thus, $D_{i}^{t}$ can be defined as follows:

(1) $$D_{i}^{t}=\max(\text{sim}(F_{+i}^{t},F_{-j}^{t}))\;\;\;,i=1\ldots N,j=1\ldots M.$$

Here, $F_{+i}^{t}$ denotes the *i*th object fragment at the *t*th frame, and $F_{-j}^{t}$ denotes the *j*th background fragment at the *t*th frame. sim(.) is the similarity measure function. By searching the most similar negative fragment with the object fragment $F_{+i}^{t}$, the maximal similarity can be obtained and is denoted by $D_{i}^{t}$. If $D_{i}^{t}$ is large enough, it means that the object fragment $F_{+i}^{t}$ is not discriminative from the background.

Uniqueness indicates whether the estimated fragment can be distinguished from other object fragments. The uniqueness $U_{i}^{t}$ is measured by the maximum difference between the estimated object fragment and other object fragments:

(2) $$U_{i}^{t}=\sum_{j=1}^{N}g(F_{+i}^{t},F_{+j}^{t},\tau )\;\;\;,i\neq j,\;\;\;i=1\ldots N$$

(3) $$g(F_{+i}^{t},F_{+j}^{t},\tau )=\left\{ \begin{array}{ll} 1 & \text{sim}(F_{+i}^{t},F_{+j}^{t})\geq \tau \\ 0 & \text{else} \end{array}\right..$$

When $U_{i}^{t}$ increases, it indicates that there are many object fragments having the similar features with the estimated object fragment. Therefore, the estimated object fragment is not unique, which is ambiguous for locating the object.

According to $D_{i}^{t}$ and $U_{i}^{t}$, the pre-selected fragments are identified by { $F_{i}:D_{i}^{t}<\tau_{1},\;U_{i}^{t}<\tau_{2}$}, where these fragments are DUFrags. The sample set of DUFrags is denoted by Ω***_DU_*** = {**F_1_**, **F_2_**, …, **F**_**N**_**DU**__} (time *t* is omitted here and below), where the sample number of Ω***_DU_*** is *N*_DU_. *τ_1_* is set according to the average discrimination capability in the worst selection for the initialization frame, and *τ_2_* is related with the number of positive fragments:

(4) $$\tau_{1}=\alpha_{1}\cdot \frac{1}{N}\sum_{i=1}^{N}D_{i}^{0},\;\;\;\tau_{2}=\alpha_{2}\cdot N,\;\;\;\alpha_{1},\alpha_{2}\in [0,1].$$

In general, unique fragments do not indicate that there is only one piece of such fragment. For example, the eyes in Lena’s face shown in Fig. 2 are likely to be both considered as unique fragments for their abundant amount of information and subtle difference. Thus, the value of *τ_2_* is often set to be greater than 2.

In fact, fragment selection is a procedure of features selection and dimension reduction. The obtained DUFrags allow the proposed algorithm to be able to perform well in distractive environments and with interference from similar objects. The computational resources are spent on more informative regions. An example is shown in Fig. 4 with *N* = 25 and *N*_DU_ = 18, where the searching time can be reduced by 28%.

### 3.2 V_DUFrags selection based on Harris-SIFT filter

In the tracking process, particle filter is used to obtain the candidate fragments at each frame, where the searching area is restrained in a small range. The small searching range is defined as a neighborhood surrounding the predicated position, which extends fragment-size pixels from predicated location along left, right, top, and bottom directions, respectively. For each DUFrags, the top ranking *N*_P_ locations with high-color histogram similarities are selected. Thus, for *N*_DU_ fragments in Ω_DU_, there are *N*_DU_ × *N*_P_ candidate fragments. These candidate fragments can obtain a set, denoted as $\Phi =\left\{F_{1}^{1},F_{1}^{2},\ldots ,F_{1}^{N_{P}},\;F_{2}^{1},F_{2}^{2},\ldots ,F_{2}^{N_{P}},\ldots ,\;F_{N_{DU}}^{1},F_{N_{DU}}^{2},\ldots ,F_{N_{DU}}^{N_{P}}.\right\}$ Then, Harris-Sift filter is used on Ω***_DU_*** and **Φ** to identify the valid DUFrags.

For a two-dimensional image, Harris corners are those points sharply changing the brightness, where they can preserve the important visual characteristic simply using a small number of corners. In this manner, the computation is efficient. Harris corners are stable in cases of translation, rotation, and noise, but Harris corner matching based on correlation can easily produce mismatching for illumination and scale changes. On the other hand, SIFT is rotational invariant, affine transformation invariant, and scale invariant. However, the conventional SIFT points simply represent the points of interest having the digital characteristic, not the visual characteristics. In addition, the number of SIFT points is seriously affected by thresholds used in SIFT. Therefore, a Harris-SIFT filter is proposed in this paper, which detects Harris corners and computes their SIFT features for matching. The purpose of Harris-SIFT filter is to filter out the invalid fragments.

In detail, Harris corners in Ω***_DU_*** and **Φ** are extracted, respectively, and their corresponding SIFT feature vectors are computed. Subsequently, the Euclidean distances of SIFT feature vectors between Ω***_DU_*** and **Φ** are calculated for matching. Random Sample Consensus (RANSAC) is used [67] to eliminate the mismatched points.

**Fig. 5:**
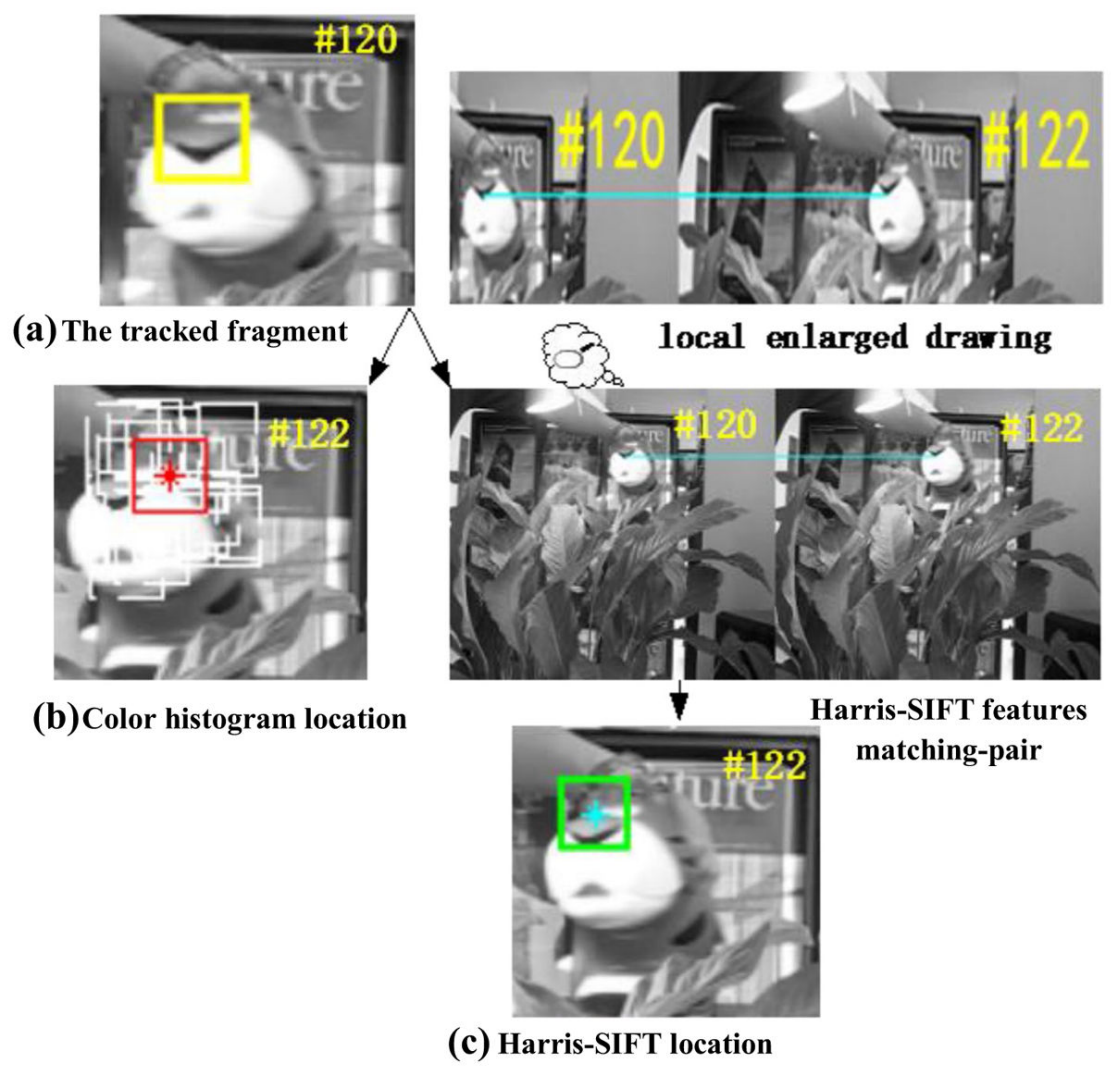
Location comparison for color histogram similarity vs. Harris-SIFT features matching-pair

In this paper, SIFT is used instead of the traditional color histogram feature. As shown in Fig. 3d, SIFT filtering determines the top row fragments to be valid ones, where correct tracking result can be obtained as shown in Fig. 3c. Apart from occlusion, an actual video with slight fuzzy, rotation, and gesture change situations is also tested to compare the effectiveness of SIFT and color histogram. The fragment marked with yellow rectangle in Fig. 5a is considered as the tracked fragment. In particle filter framework, the white rectangles in Fig. 5b show all the top ten candidates, which have higher color histogram similarities compared with the tracked fragment. The red rectangle shows the weighted location using these top ten locations. Obviously, drifting occurs and the tracking fails. In contrast, SIFT features are extracted in the regions of the top ten candidates. If there is SIFT feature matching-pair between a candidate fragment and the tracked fragment, the candidate fragment is declared as the valid fragment. Therefore, the location determined by SIFT features matching-pair is marked with green rectangle, as shown in Fig. 5c, which is apparently correct. These illustrations demonstrate that adopting valid fragment can improve the accuracy of tracking, and SIFT features matching-pair can determine the valid fragments and filter out the invalid fragments even in the presence of occlusion, rotation, slight gesture change, and noises.

### 3.3 V_DUFrags selection based on spatial constraint

**Fig. 6:**
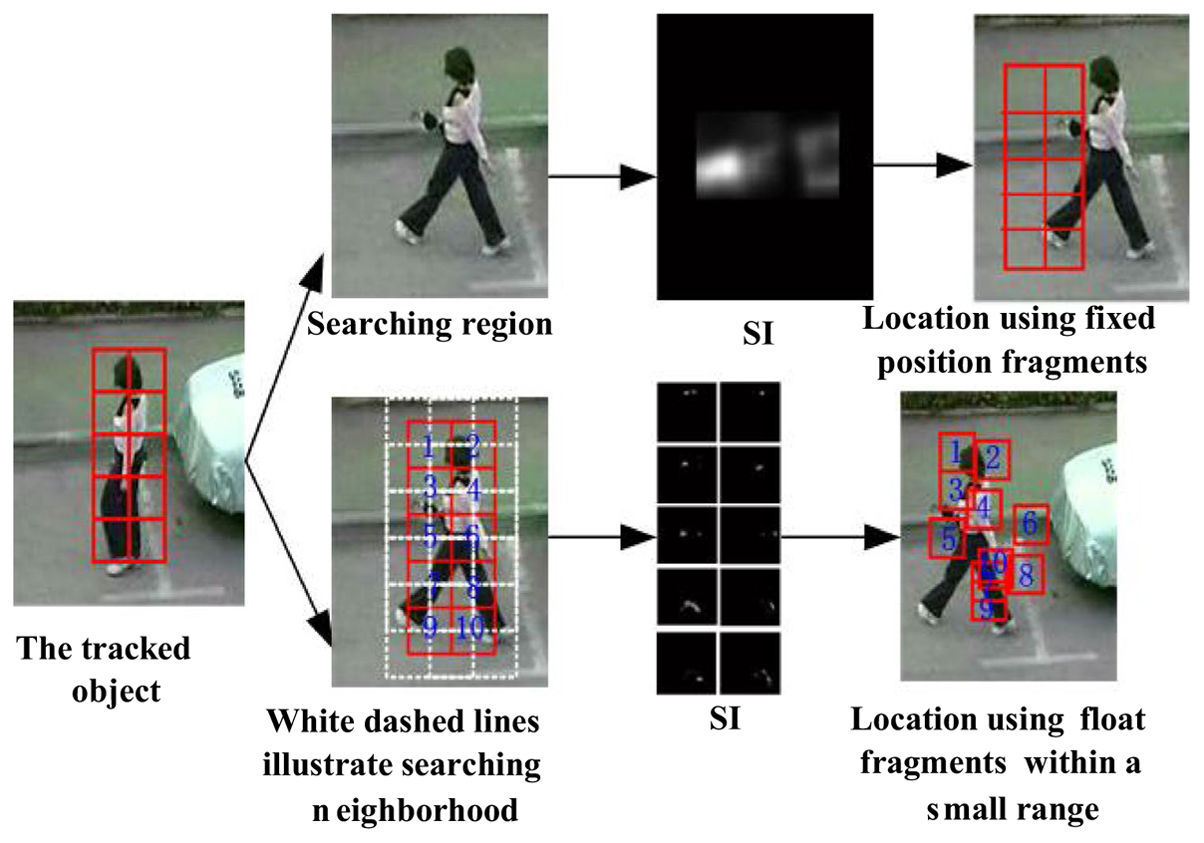
Fixed position fragments tracking and float fragments tracking within a small range

If the object undergoes scale and orientation change, non-rigid deformation, and gesture change, the position of each fragment relative to the center of the object will also change. It is reasonable to use flexible fragment grid. However, simply allowing fragments to freely float or move within a small range can easily result in mismatching. Two examples using fixed position fragments (the top row) and floating fragments (the bottom row) are shown in Fig. 6. For fragments with fixed positions, the position of each fragment relative to the center of the object is fixed. In contrast, for those floating fragments, each candidate fragment is searched within its local neighborhood, where the locations with the highest similarity of color histogram are shown for each fragment. As shown in the top row, for non-rigid pedestrian, the fragment structure of fixed position can lead to incorrect tracking result. Comparatively, if the floating fragment structure is adopted, some of the locations are basically correct (such as fragments 1–5), although the others are obviously mismatched (such as fragments 6–10). Such deformed fragments or noisy fragments sometimes completely drift from the object, which will ultimately lead to completely wrong localization of the object. It is of significant importance to eliminate the mismatching in flexible fragment grid.

It should be noticed that these correct fragment locations maintain a relatively ordered geometric structure, while the incorrect fragment locations are not in order. Therefore, spatial structure constraint among these fragments as well as the current fragments and the previous template fragments are necessary for excluding the mismatching and achieving accurate tracking. Since the existing geometric structure or topology structure constraint based on graph representation is too complicated, three simple measures are proposed to guarantee the spatial constraint in this paper. First, a fragment is allowed to move within a small range surrounding the predicted position that is estimated using a motion model [68]. Motion model prediction can adapt to objects in both rapid and slow motion, where a small range searching allows small changes of the fragment’s position, but not introducing too much interference. Small changes of the fragment’s position often correspond to small deformation, rotation of the object, which is particularly desirable for non-rigid objects. Second, SIFT matching is used to affirm the correct fragments and exclude the incorrect fragments based on highly localized features. Third, connection evaluation and structural consistency are further used to determine the correct fragments and exclude the incorrect fragments, which reflect the spatial constraint among the fragments.

**Fig. 7:**
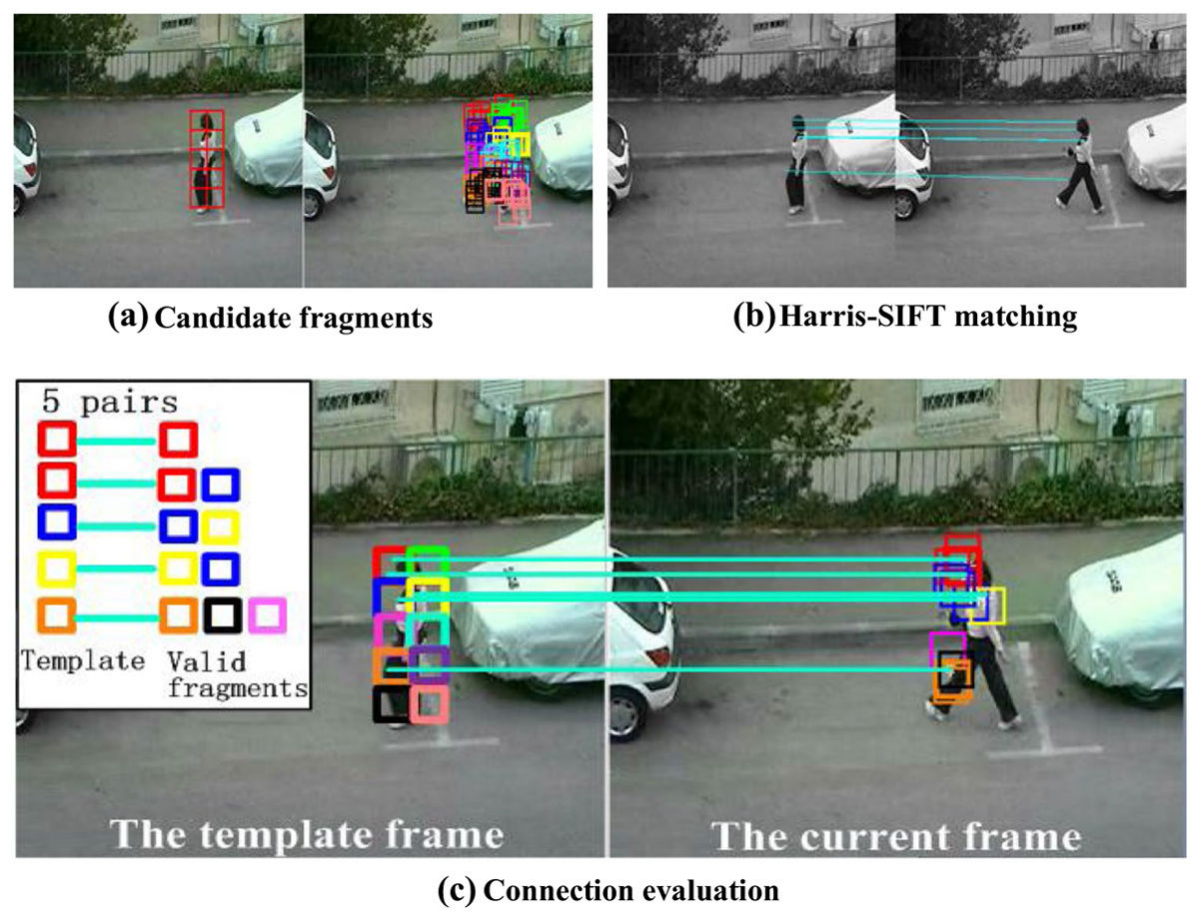
Float fragments with structure constraint

Connection evaluation requires that the valid candidate fragments in **Φ** are eight-neighbor connected with template fragment in Ω***_DU_***. For the example shown in Fig. 6, 10 DUFrags (shown with different colors, *N*_DU_ = 10) are selected depending on the discrimination and uniqueness. For each DUFrag, the proposed method searches for the candidate fragments at the current frame within a small range using a particle filter. A small range uses the previous definition. As can be seen in Fig. 7a, for each DUFrag, ten candidate fragments (*N*_P_ = 10) having top-rank color histogram similarities are found. Then, Harris-SIFT features are computed for these candidate fragments, and five matched pairs are obtained, as shown in Fig. 7b. The five fragments in Ω***_DU_*** are represented in the red, red, blue, yellow, and orange squares. According to Harris-SIFT, the corresponding matched fragments in **Φ** are red, red and blue, blue and yellow, yellow and blue, orange and black, and pink ones, as shown in Fig. 7c. It should be noted that one matched Harris corner can be covered by several different candidate fragments. An example is shown with the orange DUFrags on the person’s knee. The matched corners at the current frame are covered by two orange fragments, one pink fragment, and one black fragment. Since the four candidate fragments are all eight-neighbor connected with the original orange fragment in the template, these are all valid fragments. Connection evaluation is useful to remove those incorrect Harris-SIFT matching with erroneous spatial drifting.

**Fig. 8:**
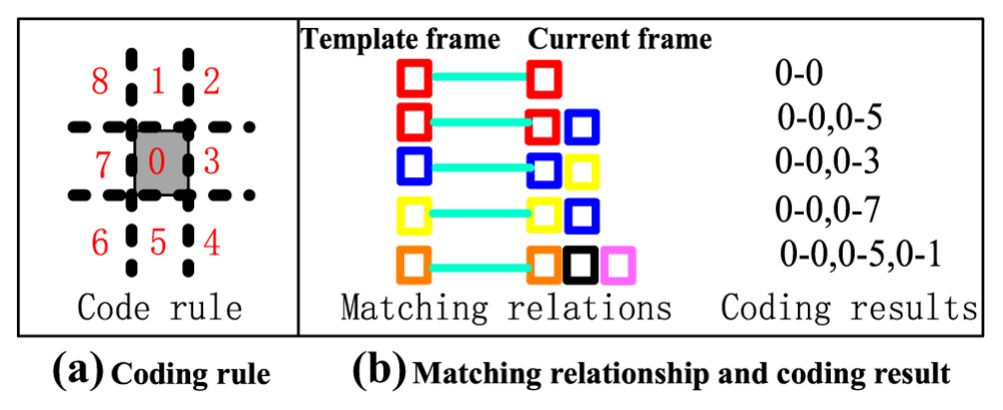
Coding rule and illustration

Structural consistency is another way to guarantee the spatial constraint. For connection evaluation, the corresponding matched fragments in Ω***_DU_*** and **Φ** along with a matching-pair are eight-neighborhood. The eight directions of eight-neighborhood can be shown in Fig. 8a. Thus, considering each fragment in Ω***_DU_*** as a center, the corresponding matched fragment in **Φ** is coded based on its relative offset direction. For example, for the matching-pairs in Fig. 7c, if the matching relationship is red–red, the coding result should be 0–0; if the matching relationship is red–blue, the blue fragment is below the red fragment and the coding result should be 0–5. The coding results are finally illustrated in Fig. 8b.

**Fig. 9:**
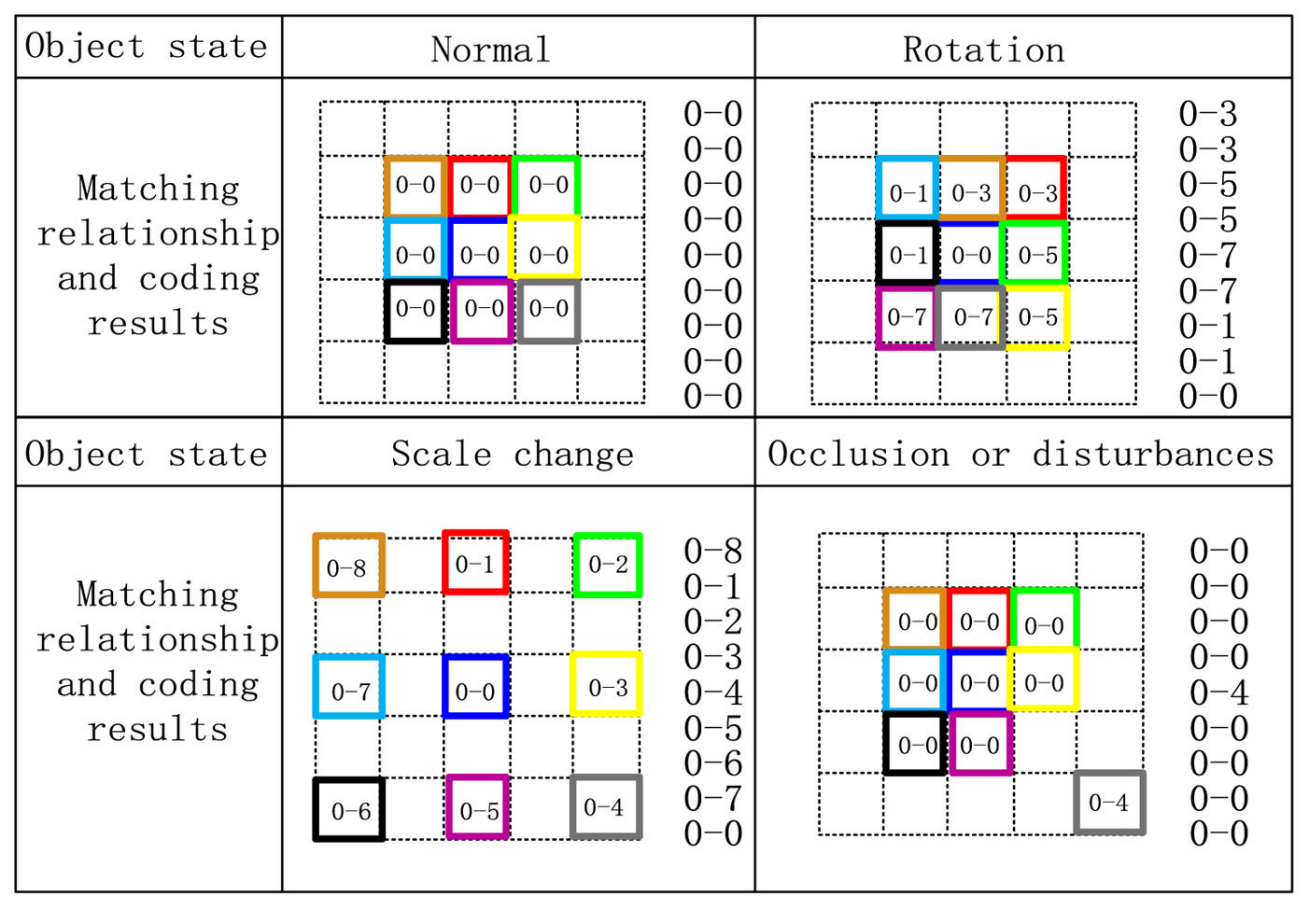
Coding results for different object states

More generally, different states, including rotation, scale change, occlusion, and similar disturbance, are simulated. The matching relations and the coding results are illustrated in Fig. 9. Refer to eight-connection, suppose, there exist nine matching-pairs, and the corresponding two matched fragments are represented by a same color. Obviously, if the object is in normal state, the coding results of all the fragments are more likely to be 0–0; if the object is in rotation state, the distribution of the coding results is more likely to be uniform; if the object is in scale change, the distribution of the coding results is also uniform and the change trend is more likely to be a roulette; if a small part of the object is occluded or disturbed, the coding results of these small regions are different from those of the coding results of those most regions, which means that a small amount of coding results do not satisfy the structural consistency.

Hence, structural consistency is proposed. A candidate fragment, whose coding result obviously does not satisfy structural consistency, is considered to be an invalid fragment. Structural consistency contains two situations. The first situation is similar to that of normal state, occlusion, or disturbance state, which indicates that a certain coding result is dominant and can be regarded as the consistent structure. The matching relations different from the consistent structure are considered to be invalid. The second situation is similar to that of rotation and scale change, which indicates that all the coding results are scattered and the distribution of all the coding results is uniform to some extent. Therefore, consistent structure does not exist and structural constraint does not work.

Therefore, the invalid fragment selection strategy based on structural consistency is summarized as follows: counting all the coding results, and recording the maximum frequency *f*_max_ and the minimum frequency *f*_min_. All the fragments which are consistent with the conditions in (5) should be treated as invalid fragments, and the remaining fragments satisfying structural consistency should be treated as valid fragments. Here, we choose 0.5 < *β* ≤ 1, 0.5 < *γ* < 1, and the specific value is determined empirically. In (5), the frequency of its coding result is *f* for the evaluated fragment:

(5) $$\text{Invalid}\;\text{Frag}=\left\{ \begin{array}{ll} 1 & if(f>\beta ,\;\text{or}\;\gamma f_{\max}<f_{\min})\\ 0 & \text{else} \end{array}\right..$$

Therefore, a candidate fragment in **Φ** is considered to be valid if there exists SIFT matching-pair and connectivity relation between this fragment and its corresponding object template fragment in Ω***_DU_***, as well as structural consistency for the fragments retained in **Φ**. All the valid fragments in **Φ** are denoted as **Φ*_V_DU_***, and all the matched valid fragments in Ω_DU_ are marked as Ω***_V_DU_***. **Φ*_V_DU_*** keeps the stable and structured parts for the object.

### 3.4 Object location based on V_DUFrag fusion

After Harris-SIFT filtering, all the fragments in Φ_V_DU_ are used to determine the object location. Suppose that the SIFT matching points considered at the current frame are {*P*_1_, *P*_2_, …, *P*_L_}, and these points existed in the fragments {**F_1_, F_2_,** …**, F_L_**}, respectively. The object tracking likelihood function using multiple fragments can be defined as:

(6) $$\hat{P}(T)=\sum_{n=1}^{L}P(T|P_{n})P(F_{j}),\;\;\;P_{n}\in F_{j},1\leq j\leq L.$$

The mode of *P*(**T**|*P_n_*) denotes the localization result using *P_n_*, and *P*(**F_j_**) denotes the belief of ***F_j_***, which is considered as the weight of *P*(**T**|*P_n_*). For *F_j_*, suppose that its corresponding template fragment is $F_{i}^{\mathrm{Temp}}$, which is denoted as $F_{i}^{\mathrm{Temp}}\to F_{j}$. The corresponding SIFT matching corners are denoted as $P_{m}^{\text{Temp}}\to P_{n}$. It should be noted that $P_{m}^{\text{Temp}}\in F_{i}^{\mathrm{Temp}}$, *P_n_*∈ **F_j_**, which indicates that the position is in the region of the fragment.

**Fig. 10:**
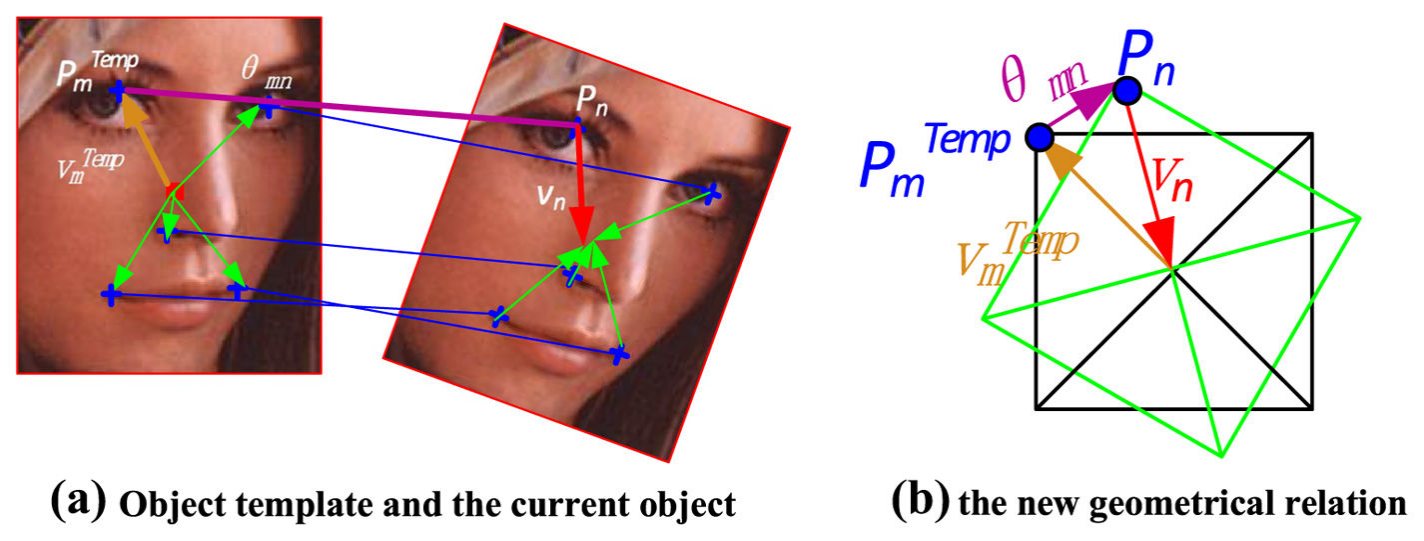
Geometrical relation location illustration

Each *P_n_* can decide one current object center *P*(**T**|*P_n_*), and our aim is computing the new geometrical relation between *P_n_* and *P*(**T**|*P_n_*), which is denoted as *v_n_*. The processing is illustrated in Fig. 10. *v_n_* is determined by two terms, one is the recorded geometrical relation between $P_{m}^{\text{Temp}}$ and the template object center denoted as $v_{m}^{\text{Temp}}$, and the other is the geometrical relation between $P_{m}^{\text{Temp}}$ and *P_n_* denoted by *θ_mn_*. It can be obtained as follows:

(7) $$v_{n}=v_{m}^{\text{Temp}}+\theta_{mn}.$$

Thus, each *P_n_* in **F_j_** can be used to identify one object center. Based on (6), several matching corners at the current frame can be used to localize the final object. Here, the frequency of coding result and the color similarity between $F_{i}^{\mathrm{Temp}}$ and **F_j_** are combined to evaluate the belief of **F_j_**. As a voting process, all the SIFT matching corners in **Φ*_V_DU_*** are used to obtain a likelihood function with their locations, similarities, and spatial constraint.

Since one matched Harris corner can be covered by several different candidate fragments, several **F_j_** may have a substantial spatial overlap in images such as the example shown in Fig. 7c, where their beliefs may be correlated with each other.

### 3.5 Feature fusion update

Object drift is a common problem in object tracking, largely caused by imperfect template updating. A common strategy to determine the necessity of updating template is by comparing the feature similarity between the best-matched candidate and the template. However, this update strategy does not always perform well in the cases that the similarity measure is not reliable. For instance, color histogram is prone to error due to occlusion, deformation, similar background, and noise disturbance. Moreover, the threshold of similarity for making updating decision is not easy to be determined.

In this paper, we set a rule that the template should not be updated in the cases of occlusion or large deformation. Otherwise, it can be updated in cases of zoom in/out or rotation. It should be noted that Harris-SIFT and structural consistency, which is used to determine the V_DUFrags in our method, are sensitive to occlusion but can provide accurate information on rotation and scale change. Therefore, an object template update strategy is proposed which is based on Harris-SIFT, structural consistency, and color histogram measures. More specifically, the template updating condition is described as follows:

#### Update condition

Let us consider each candidate fragment determined by color histogram at the current frame. If there is an SIFT matched-pair in the fragment and the fragment maintains a dominant structural consistency or a uniform coding result compared with other fragments, the fragment is considered to be in updating state. If all the fragments of the object are in updating state, the update condition is satisfied. This condition usually indicates that the object is not in partial occlusion or serious deformation, but rotation and scale change from a certain extent are permitted.

#### Scale estimation

**Fig. 11:**
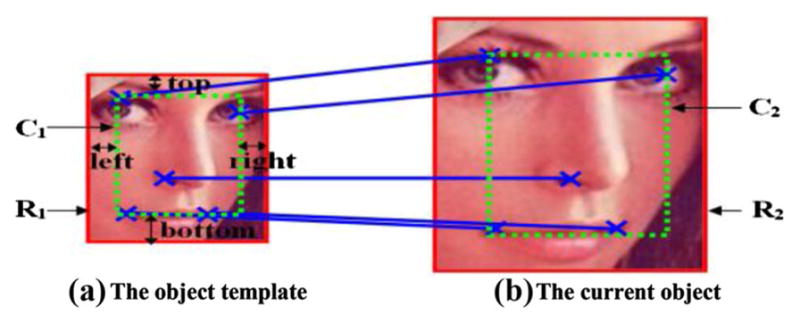
Scale estimation based on SIFT matching-pairs

In the proposed algorithm, scale change is estimated based on available SIFT matching-pairs. This strategy is illustrated in Fig. 11. First, the bounding boxes of the SIFT matching corners in the object template and the current frame are defined as **C_1_** and **C_2_**, respectively, and the bounding box of object template is defined as **R_1_**. Second, the distance between **C_1_** and **R_1_** is calculated, which is denoted as Gap_1_ = (top, bottom, left, right). It can be obtained as follows:

(8) $${\text{Gap}}_{2}=\sqrt{\frac{\text{Area}\;(C_{2})}{\text{Area}\;(C_{1})}\times {\text{Gap}}_{1}}.$$

Finally, by adding Gap_2_ to **C_2_**, the bounding box of the object in the current frame can be obtained, denoted as **R_2_**.

For each tracking frame, scale estimation will be performed once the update condition is satisfied, and the object template is also updated. Afterwards, DUFrags are chosen again.

Based on the above principle, the proposed Structured Fragment-based Tracking (SFragT) method can be summarized in Algorithm 1.

**Algorithm 1:** Structured Fragments-based Tracking(SFragT)

**Input:**
Object position in the first frame: *P*
**Select** DUFrags.
Fragment Model: Ω*_pos_*={F_+1_^t^, F_+2_^t^,…, F_+N_^t^}, Ω*_Neg_*={F_−1_^t^,F_−2_^t^,…, F_−M_^t^}
Target: **Ω*_Du_*={F_1,_ F_2_…, F_N_DU__}**
**Output:**
Object position in the current frame: *P*
1.**while**(frame!=NULL) **do**
2.**Select** V_DUFrags. Apply Harris-SIFT filter on **Ω*_Du_*** and **Φ** to generate **Ω*_V_Du_***, and **Φ*_V_Du_***.
3.**Confirm** V_DUFrags. Based on spatial constraint, including connection evaluation and structural consistency to confirm **Φ*_V_Du_***
4.**Track**. The object is located with V_DUFrags according to Eq.(6)
5.**Learn**. A new appearance model with the selected fragments in update condition.
6.**end while**

## 4 Experimental results

In this section, DUFrags and V_DUFrags are first qualitatively tested to demonstrate the effects for tracking. And then, the tracking results with qualitative and quantitative evaluation on OTB 2013 [69] data sets are presented. Finally, computational cost and limitations are also analyzed.

### 4.1 DUFrags and V_DUFrags Selection

**Fig. 12:**
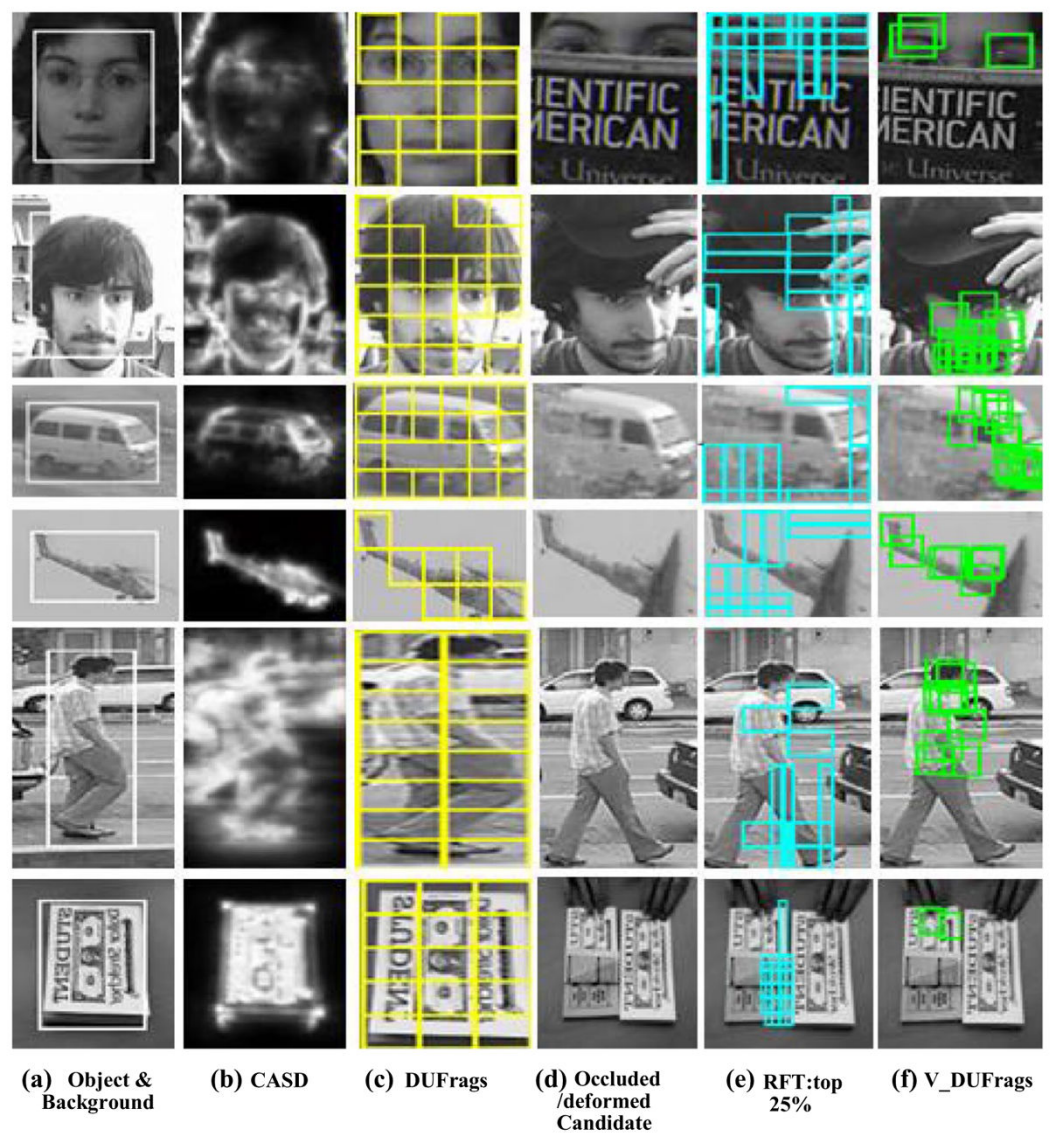
DUFrags and V_DUFrags Selection

Detecting the discriminative and unique fragments as well as occluded or deformed fragments of the object are the important steps for the proposed tracking algorithm. Figure 12 shows some results of DUFrags and V_DUFrags selection. The test images derive from the tracking benchmark data set. In these images, some objects have similar regions in themselves, and some objects have similar regions with their background. In the corresponding video sequences, occlusion, deformation, pose changes, and similar disturbance occur.

Figure 12a shows the object and its neighboring background, and Fig. 12c shows DUFrags found by the proposed pre-selection method (labeled by yellow rectangles). As the existing fragment-based methods seldom include pre-selection mechanism considering visual distinct fragments, context-aware saliency detection (CASD) [33] results are illustrated for reference. Similar to the proposed method in this paper, CASD does not need prior knowledge or training database, and the detected saliency regions (shown as bright areas in Fig. 12b) are based on local low-level consideration, global considerations, visual organizational rules, and high-level factors. The experimental results show DUFrags and CASD are well coincided with each other not only for high discrimination regions, such as eye, nose, and mouth, but also for low uniqueness regions, such as hair and cheek in face images. Especially, when the background is simple, such as plane and dollar images, CASD and DUFrags both behave the similar results. However, when the background is complicated, such as walker images, CASD is not exclusive to the object; the leg is even darker than some neighboring background, while DUFrags regard all the object fragments as discriminative and unique fragments. When the image is noisy, such as minibus image, CASD ignores the car roof, window, and wheel, while DUFrags appear to be tracking relevant. In addition, DUFrags remove the background features which might be included in the object bounding box.

Furthermore, for V_DUFrags selection, the fragment selection method in Robust Fragment-based Tracking (FragTrack, RFT) [37] is used for comparison, because it is similar to our algorithm. In RFT, the object is divided into 36 overlapping fragments and those with top 25% ranked similarity are selected to represent the current candidates. The color histogram is used in similarity measure. As shown in Fig. 12e, some occluded fragments or background fragments are selected incorrectly by RFT, especially when the occlusions are large or have the similar color as the object, while some useful fragments are ignored. On the contrary, the proposed Harris-SIFT filter can filter out occluded or highly deformed fragments. A typical example is shown by the dollar images. The tracked object undergoes great deformation and disturbance by a similar object. The proposed SFragT method is able to capture the corrected fragments of the tracked dollar bill. These examples demonstrate that overall V_DUFrags are robust and reliable to describe the tracked objects.

**Fig. 13:**
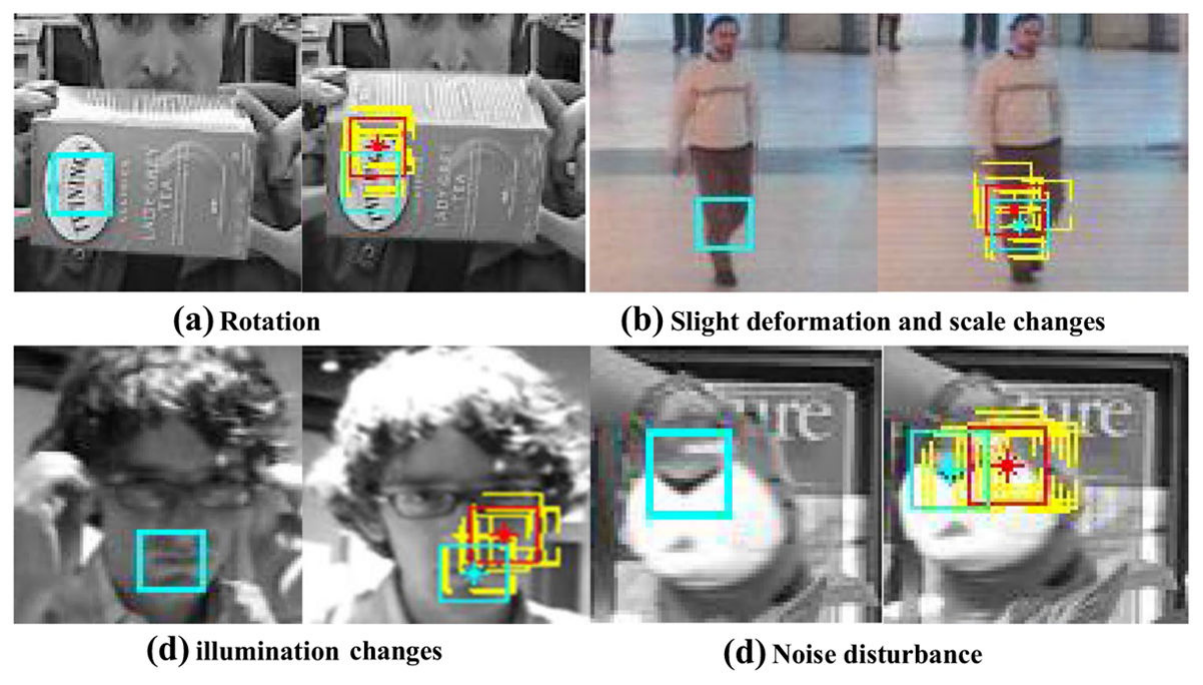
Robustness of Harris-SIFT filter illustration

Besides the strong capability to exclude occluded or highly deformed fragments, Harris-SIFT feature is robust to rotation, scale changes, slight deformation, illumination changes, and noise disturbance. Four illustrative examples are shown in Fig. 13 to demonstrate the effectiveness. For each example, the cyan square in the left image shows the template fragment, and in the right image, the yellow boxes indicate ten best candidates found by a particle filter using color histogram similarity, and the red box shows the weighted average location of them. As can be seen, while a drifting problem occurs to particle filtering results, valid fragments determined by Harris-SIFT filter(cyan box) are correct in these situations. The examples also illustrate that Harris-SIFT has highly localized characters.

### 4.2 The stability of V_DUFrags selection

**Fig. 14:**
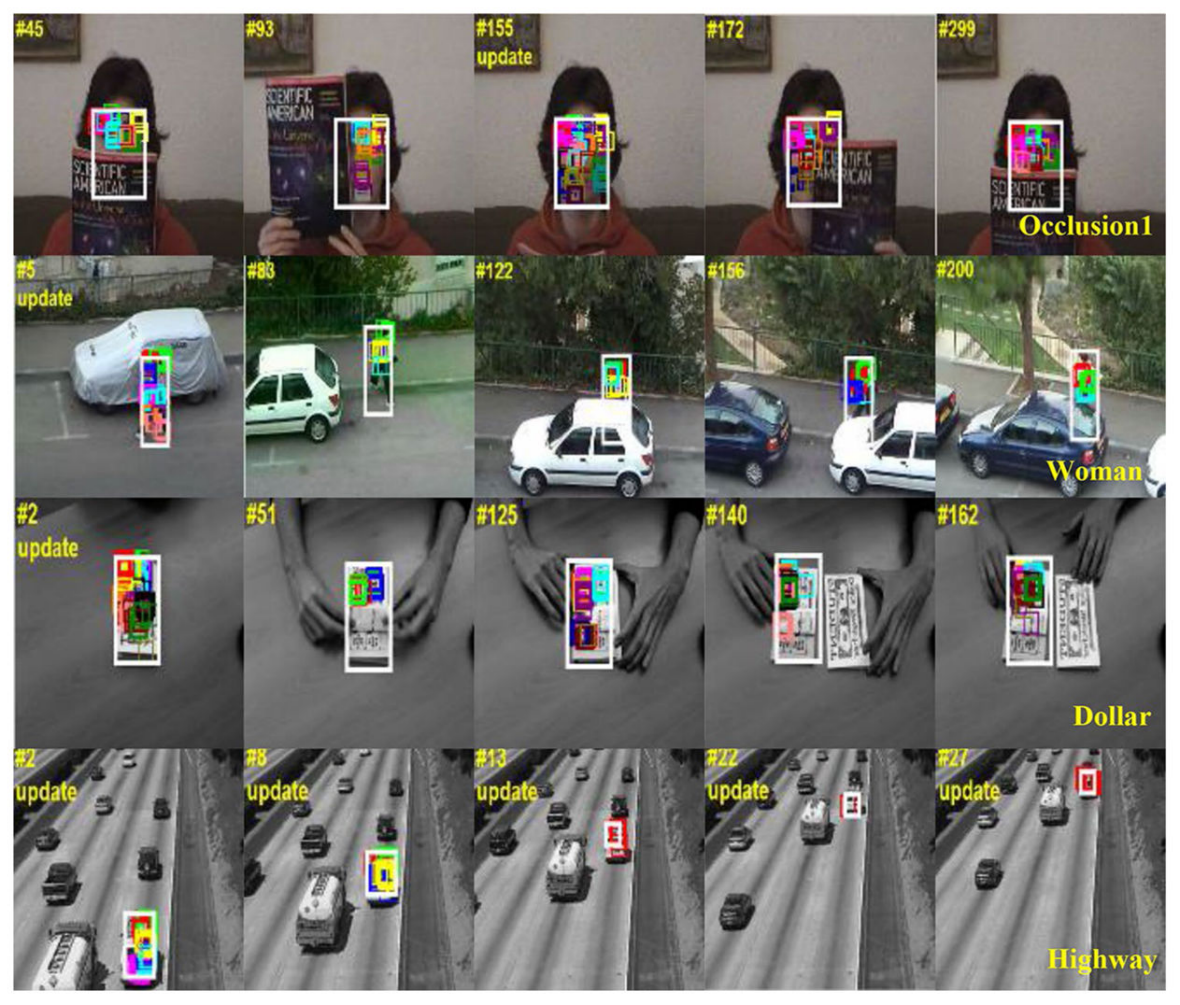
Stability of V_ DUFrags

To verify the stability of V_DUFrags selection, four video sequences including partial occlusion, deformation, similar disturbances, and scale change are tested. Figure 14 displays the results for some intermediate frames. The color squares indicate valid fragments **Φ*_V_DU_*** Confirmed by the proposed algorithm, and white rectangles indicate the object location using **Φ*_V_DU_***.

For occlusion and deformation, such as occlusion1 sequence, woman sequence, and dollar sequence, the proposed algorithm can adaptively select those unoccluded or stable fragments. Once the object returns to unoccluded state, V_DUFrags can again cover the whole object as much as possible (see #155 frame in Occlusion1 sequence). It is worth noting that the update does occur for the normal state.

Flexible fragment grid under floating range, spatial constraints, and Harris-SIFT matching ensures flexibility to slightly deformed objects and prevent fragment from deviating away too much. Hence, V_DUFrags are almost always kept within the object region and the position relationship among each fragment is basically stable. For example, compared to the results in Fig. 7, the green V_DUFrags in woman sequence are always concentrated in the upper part of the object, and the occluded or deformed incarnadine V_DUFrags are disappeared.

The highway sequence demonstrates our algorithm’s capability to adapt to scale change. The object is decomposed into four fragments at the first frame. According to update condition evaluations and scale estimation results, the object template is updated multiple times. By frame #13, the object is represented by only one fragment.

### 4.3 Tracking results

#### 4.3.1 Experimental setup and evaluation metrics

We test our tracker on OTB 2013 visual tracker benchmark [69] (with 50 fully annotated videos, more than 29,000 frames). This benchmark divides all the videos into 11 categories based on their attributes, including illumination variation (IV), scale variation (SV), occlusion (OCC), deformation (DEF), motion blur (MB), fast motion (FM), in-plane rotation (IPR), out-of-plane rotation (OPR), out-of-view (OV), background clutters (BC), and low resolution (LR). The main attributes of all the experimental sequences can refer to [69]. The proposed SFragT tracker is compared with other state-of-the-art trackers, including DFT [70], LOT [6], CT [11], Struck [18], KCF [22], and Staple [36], which achieve good results on the benchmark. Note that, particularly, KCF and Staple are basically the top trackers at present. Note that all the plots are generated from OTB 2013, and the tracking data of KCF and Staple are from the authors.

Two measurements, precision plot and success plot [71], are adopted in this paper to compare the performance of different methods. The first metric, which represents the precision rate, indicates the percentage of frames whose tracked locations are within the given threshold distance to the ground truth. In this paper, a representative precision score with the threshold equal to 20 pixels is used to rank the trackers. The success plot is employed as another metric. It is based on overlap ratio, which is defined by formula (9):

(9) $$\text{rate}=\frac{\text{Area}\;(\mathrm{RT}\cap \mathrm{RG})}{\text{Area}\;(\mathrm{RT}\cap \mathrm{RG})},$$

where, **RT** is the tracked bounding box and **RG** is the ground-truth bounding box. The success plot illustrates the percentage of frames, whose overlap ratios are larger than the given threshold *t*_o_, *t*_o_ ∈ [0,1]. The area under the curve (AUC) of each success plot serves as the second measure for ranking. We present the results of one-pass evaluation (OPE) based on the average precision and success rate given the ground-truth target state in the first frame.

#### 4.3.2 Parameters analysis

According to Table 1, the primary parameters include the size of fragment—*w* and *h*, the number of negative samples—*M*, the number of particles for each fragment—*M*_P_, the selected top-rank locations using color histogram similarities—*N*_P_, the thresholds for discrimination and uniqueness—*τ_1_*, *τ_2_*, and the thresholds for structural consistency—*β*, *γ*. In addition, there are some parameters related to Harris corner extraction and SIFT computation. These parameters are analyzed as follows.

As mentioned earlier, the size of the fragment is related to the effectiveness of color histogram similarity, and it is set to 20 pixels (*w* = 20, *h* = 20) in this paper. In fact, the size of fragment is set to 20 [46] or is set to 22 [72] in the recent literature. To verify the effectiveness of the fragment’s size, a tracking method based on color histogram is tested, and the test sequences cover many complex conditions, including OCC (Faceocc1, Walking2, plane), SV (Breadcar, Walking2), DEF (pets), FM (Highway, Highway2, Highway3), IV (Sylvester), IPR (Sylvester), OPR (Sylvester), LR (Walking2), etc. The results in Table 2 demonstrate that the size of fragment set to 20 is better for most cases. Because the partition not only can describe the meaningful color histogram feature, but also generate enough fragments for spatial representation. Consequently, the tracking results are more accurate.

The number of negative samples *M* is related to the size of the object to be tracked, and the background fragments corresponding to these negative samples should cover the neighborhood around the object as much as possible. Thus, the mapping relationship between the number of negative samples and the neighborhood area can be established. Since the negative samples are randomly sampling in the neighborhood area, it is optimal to establish a linear mapping relationship between the neighborhood area and the number of negative samples, which is represented by *M* = *k* × Area (background). Area (background) denotes the area of the neighborhood. The value of parameter *k* is set to 0.01 in this paper.

**Fig. 15:**
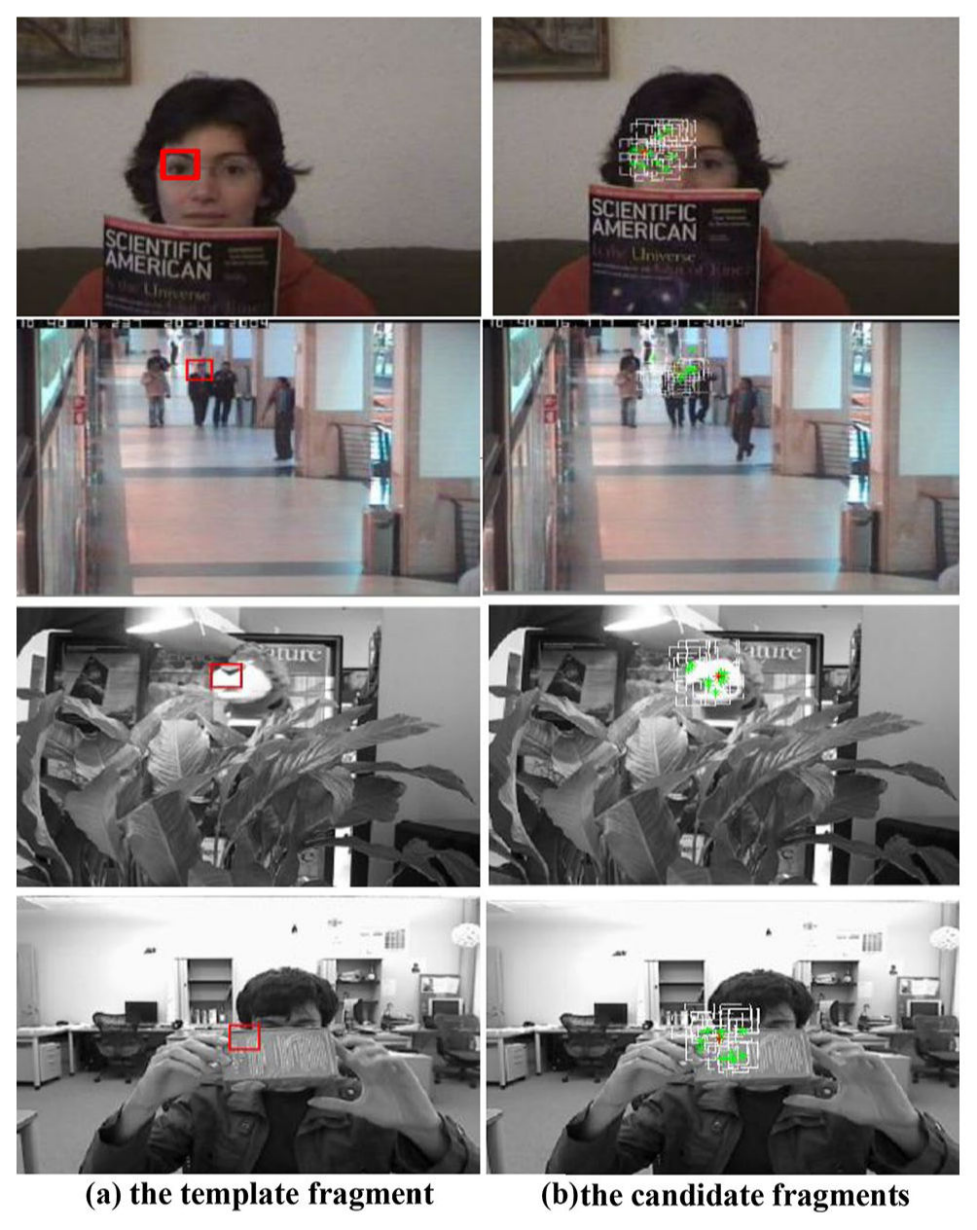
Position coverage illustration based on *M*_p_ and *N*_P_ parameters

For each fragment, there are *M*_P_ randomly sampled particles. Based on the *M*_P_ locations, the *N*_P_ top-rank locations using color histogram similarities are selected. Particles and color locations just provide the candidate fragments in this paper; therefore, as long as the locations of color histograms can cover the correct object position, the subsequent Harris-SIFT filtering can be carried out smoothly. Therefore, four scenarios shown in Fig. 15, including normal state, SV, DEF, FM, LR, IV, MB, IPR, OPR, and OV, are tested to demonstrate the position coverage. In Fig. 15a, the red rectangle denotes the template fragment, while in Fig. 15b, the white fragments denote the *M*_P_ particle positions, and the green asterisks indicate the centers of the top-rank *N*_P_ objects determined by color histogram similarity. For good observation, *M*_P_ = 20, *N*_P_ = 10. Clearly, even undergoing rapid motion, scale changes, rotation, etc., the rectangles corresponding to the green asterisks can always cover the correct position of the tracked fragment. Although more particles are helpful to improve the robustness of the tracking algorithm, considering the computation for multiple fragments as well as the search area is limited to the neighborhood of the object’s prediction position, *M*_P_ = 100 and *N*_P_ =10 are enough to adapt to various complex situations.

The thresholds for discrimination and uniqueness—*τ_1_*, *τ _2_* (equivalent to *α*_1_, *α*_2_), and the thresholds for structural consistency—*β*, *γ* are all related to the fragment selection. There are two extreme selecting cases based on the four parameters, i.e., all the fragments are selected as V_DUFrags, or no V_DUFrags. If it is the first case, the tracker is degenerated as a Harris-SIFT tracker, while if it is the second case, the tracker may fail to track target. Based on OTB 2013 database, the different parameter values are adopted to verify the influences. For each parameter, its value is limited to the own range, respectively, but can be adjusted by a fixed step length. From the theoretical analysis, because the determination of these parameters is related to object size, image characteristics, etc., the different values should be adopted in different video sequences to get the best results. The experimental results also prove this notion. Thus, based on the overall evaluation, the combinations of different parameter values are tried, and finally, a set of fixed parameter values are adopted in the experiment. That is, *α*_1_ = 0.3, *α*_2_ = 0.2, *β* = 0.8, and *γ* = 0.8.

In addition, the parameters related to Harris corner extraction and SIFT computation are determined by the traditional settings.

#### 4.3.3 Quantitative comparisons

Tables 3 and 4 show the performance of our proposed SFragT tracker in different challenges, as well as the overall performance compared with six state-of-the-art trackers. Tables 3 and 4 summarize the scores of the precision plot and success plot of each tested tracker on the 11 considered attributes, respectively. In Tables 2 and 3, the top three methods in each attribute are denoted by different fonts: bold, italics, and bold italics.

**Fig. 16:**
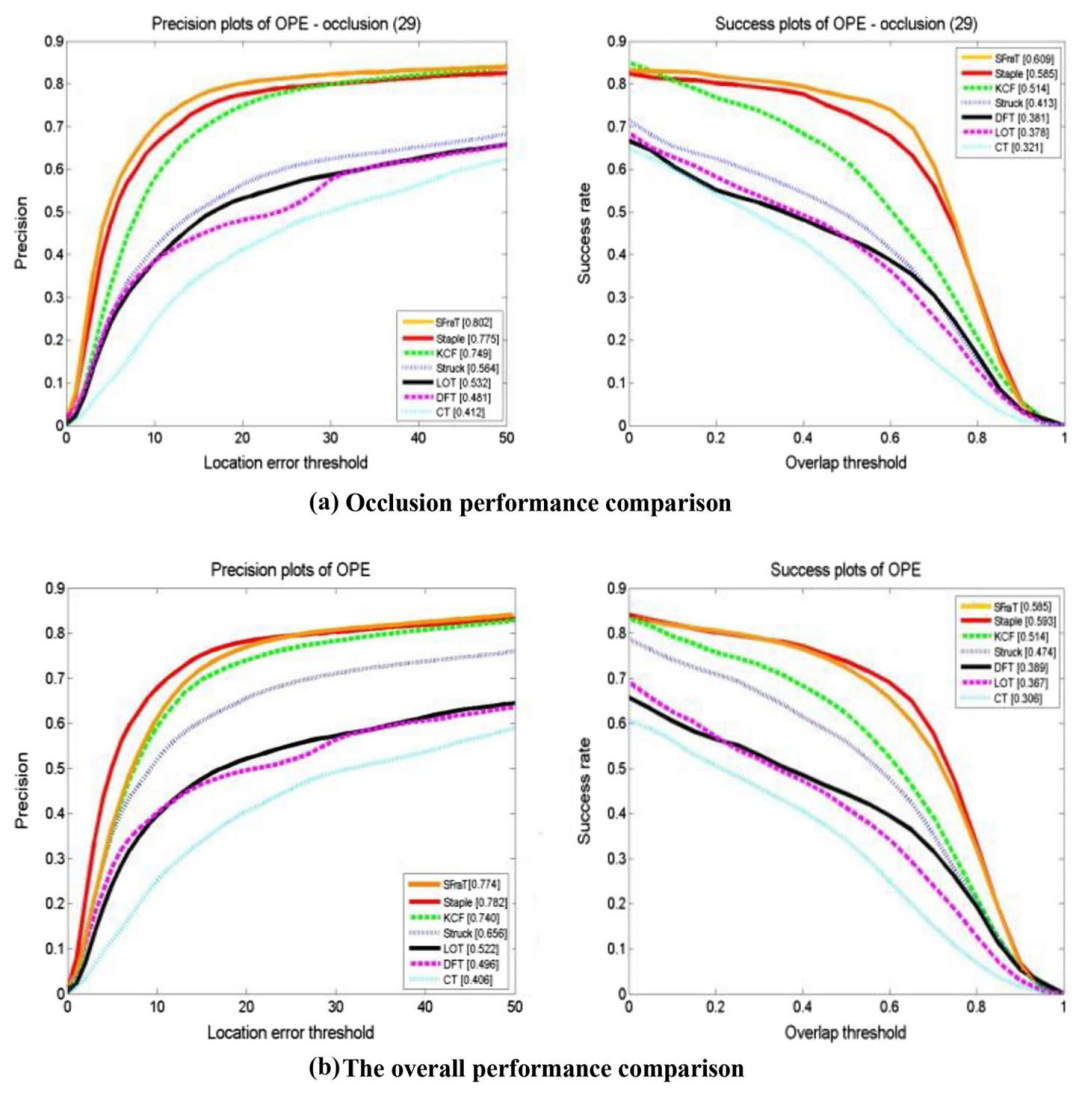
Success plots and precision plots of OPE for seven trackers

Figure 16 is the overall performance comparison, and the performance comparison on OCC. The orange curves denote the proposed SFragT tracker.

For overall performance, our proposed SFragT tracker achieves a score of 0.774 in the precision plot, which outperforms the KCF tracker (0.740), by 3.4%. In terms of success plot, our tracker achieves an AUC of 0.585, outperforming the KCF method (0.514) by 7.1%. Although SFragT ranks second place compared with Staple in the overall performance, SFragT ranks first based on both precision plot and success plot in OCC, IV, and SV categories. From the perspective of precision plot, we can conclude that in OCC category, SFragT outperforms Staple tracker by 2.7%; in IV, SFragT outperforms Staple tracker by 2.1%; in SV, SFragT outperforms Staple tracker by 0.2%. From the perspective of success plot, we can conclude that in OCC category, SFragT outperforms Staple tracker by 2.4%; in IV, SFragT outperforms Staple tracker by 0.2%; in SV, SFragT outperforms Staple tracker by 0.3%.

In terms of success plot, SFragT tracker ranks as the top tracker for 3 of the 11 attributes, and it outperforms KCF tracker on 9 attributes. SFragT tracker with motion blur and background clutters’ attribute ranks third place, mainly because SFragT cannot extract accurate Harris-SIFT feature in motion blur situation, and cannot select discriminative feature in background clutters situation.

The tracking results on those challenging sequences show that the proposed SFragT tracker is among the top 2 in overall performance, and the top 1 in terms of certain categories based on average center location error and the average overlap rate.

#### 4.3.4 Qualitative comparisons

**Fig. 17:**
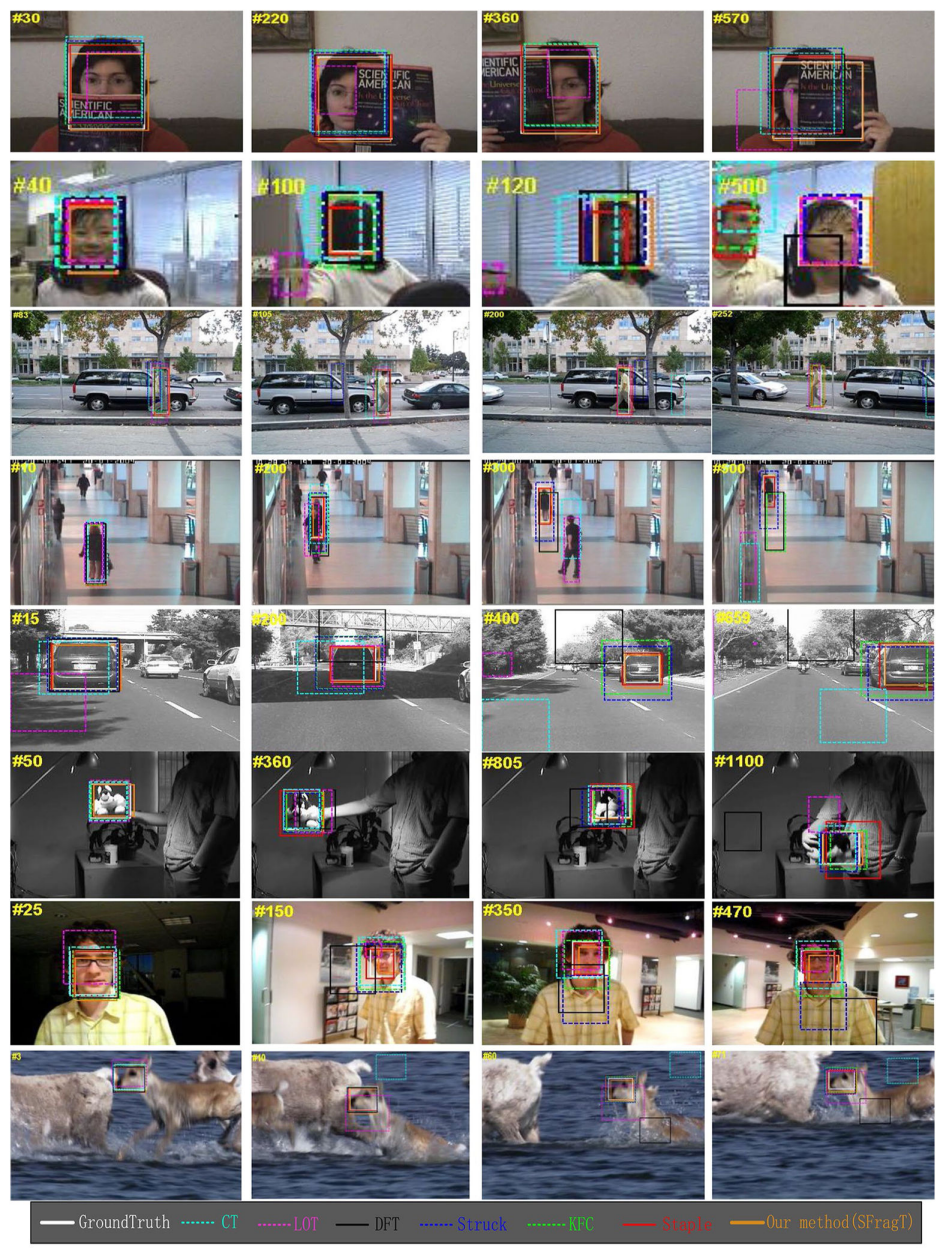
Sample tracking results for challenging sequences

Figure 17 presents screenshots of the tracking results in eight test videos where the objects suffer from different challenges. In the FaceOcc1 sequence, the face of a woman is heavily occluded by a book from the bottom, the left side, and the right side. Most of the other six compared trackers perform well on this sequence, but the LOT tracker drifts to the background. The girl video undergoes the challenges of SV, OCC, IPR, and OPR. After scale variations and out-of-plane rotations, only Struck, KCF, Staple, and the proposed SFragT tracker can track the object. When the object is heavily occluded by a man, the Staple and KCF drift to the man mistakenly, while SFragT locates the object accurately. In the David3 sequence, which contains OCC, DEF, OPR, and BC attributes, Struck, DFT, and CT trackers arise large position offset, while SFragT is more accurate in comparison. It is reasonable to believe that the proposed SFragT tracker, which uses Harris-SIFT filter to remove the invalid fragments, is more robust to occlusion.

In the Walking2 sequence and Car4 sequence, scale change is obvious. Meanwhile, Walking2 sequence has LR attribute, and the object is occluded sometimes; IV occurs in Car4 sequence. The results show that SFragT can update the object in time to adapt to scale variations, and ensure the correct tracking. Because color feature is robust to the deformation and illumination changes, and Harris-SIFT feature is rotation invariant, as well as highly positioning characteristics, SFragT is robust to IV, DEF, and rotation. The results of Sylvester sequence and David sequences also support the conclusion.

In addition, with the motion prediction and position constraint, SFragT tracker can also adapt to fast motion such as the results of Deer sequence.

From the exemplar results shown in Fig. 17, it can be observed that the proposed SFragT tracker is among the best methods, as it tracks objects with the highest accuracy for most of the sequences. Especially, in case of occlusion, large-scale change, and illumination variation, SFragT produces much better results than all other trackers. Furthermore, it can be concluded that SFragT is suitable for subtle non-rigid object, not for those severe deformation. In addition, if the object endures complex background or blur image, the tracker is easy to drift.

**Fig. 18:**
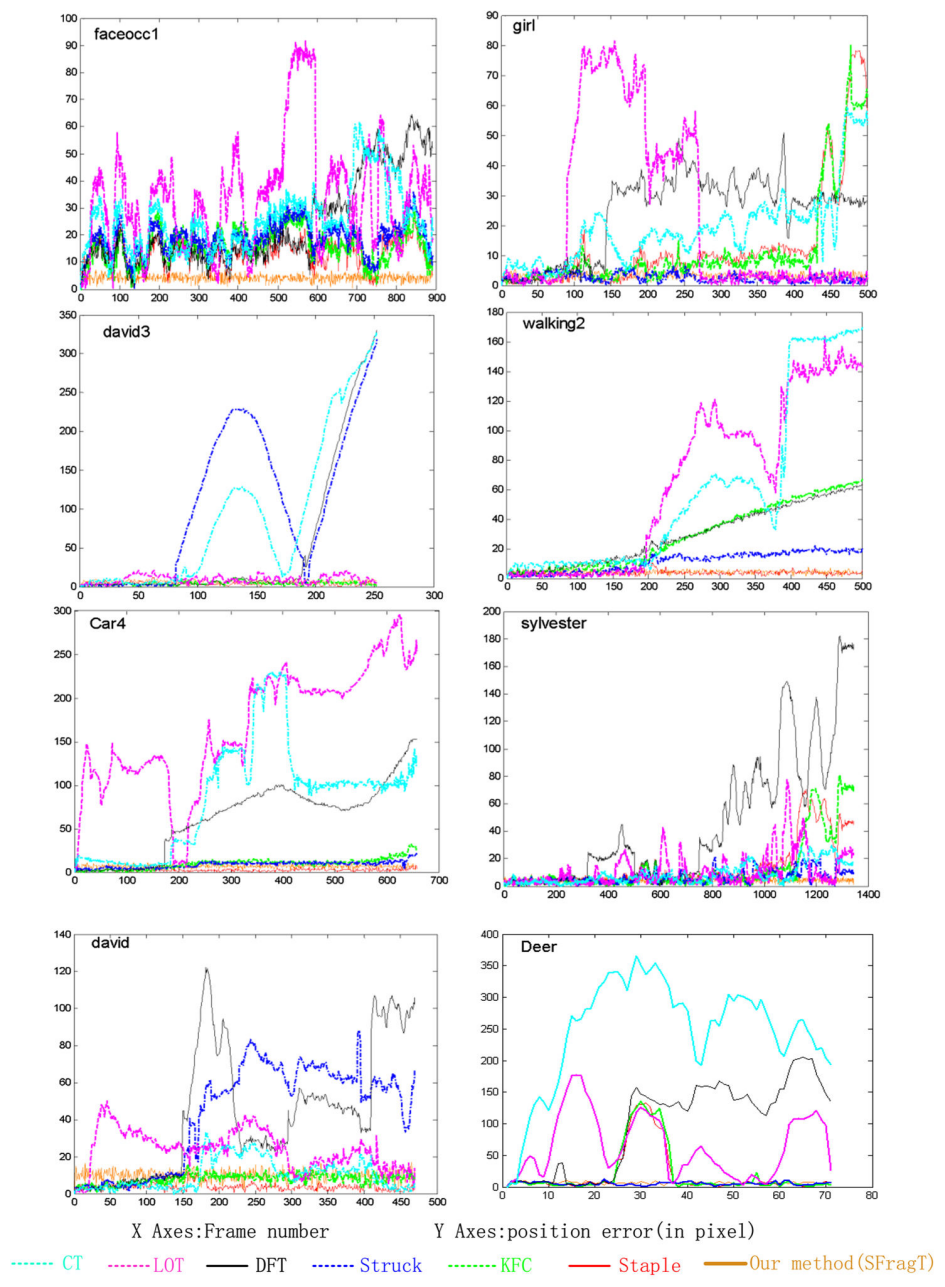
Center location error (in pixels) for challenging sequences

The location error plots corresponding to Fig. 17 are shown in Fig. 18. Consistently with results shown in Fig. 17, it can be seen that our method was among the best ones.

#### 4.3.5 Computational cost

The proposed algorithm mainly includes DUFrags selection, V_DUFrags selection, and object localization steps. DUFrags selection is carried out only when the template is updated, and it includes: (1) computing the color histogram for *N* positive samples and *M* negative samples. Suppose that the color histogram is computed using *d* dimensional feature vectors, and the size of fragment is *h* × *w*, the cost for computing the color histogram for the (*M* + *N*) fragments is O[*d* × *h* × *w* × (*M* + *N*)]. (2) Computing the similarity between *N* positive samples and *M* negative samples. The cost to compute the distance between two fragments using the Bhattacharyya distance is O(*d*^3^), so the cost for *M* × *N* times is O(*M* × *N*×*d*^3^). (3) Compute the similarity among *N* positive samples. The cost is O(*N*^2^ × *d*^3^).

V-DUFrags selection includes four steps: (1) for each DUFrag, suppose producing *M*_P_ candidate fragments using particle filter. For *N*_DU_ × *M*_P_ candidate fragments, compute their color histogram. The cost is O(*d* × *h* × *w* × *N*_DU_ × *M*_P_). (2) Extracting Harris corners in **Ω*_DU_*** and **Φ**, the cost is O(**Ω*_DU_*** + **Φ**). Suppose that the number of the Harris corners in **Ω*_DU_*** and **Φ** is *N*_H1_ and *N*_H2,_ respectively, and the dimension of SIFT feature is *d_s_*. Compute their SIFT features, and the cost is relative with the number of the Harris corners, and can be represented as O((*N*_H1_ + *N*_H2_) × *r*^2^ × *d_s_*), *r* is the radius of SIFT features. (3) SIFT features matching and RANSAC. The cost is $O((N_{H1}+N_{H2})\times d_{s}^{3})$ for SIFT features matching. Suppose that the number of matching-pairs is *N_m_*, and the cost is *S* × O(*N_m_*), $1\leq S\leq C_{N\cdot m}^{3}$. Thus, for the worst situation, the cost is $O(N_{m}^{4})$ for RANSAC. (4) Spatial constraints. The cost is O(*N_m_*).

Object localization is a weighted-sum process, and it is very fast. The computational cost is relative with the number of V_DUFrags. Suppose that *L* is the number of V_DUFrags in Φ_V_DU_, the cost for object localization is O(*L*).

Taking the aforementioned costs together, the total cost of the proposed procedure is obtained. This is the longest processing time for one frame, but not for each frame. Because DUFrags selection is not carried out for each frame.

The proposed tracking algorithm was implemented in Matlab 2013 code, and was tested on a PC with an AMD Athlon(tm) II X4 635 Processor (2.9 GHz) and 4 GB RAM. For the aforementioned video sequences, the processing time of each sequence is listed in Table 5. The processing time of the algorithm mainly spends on color similarity computation in particle filter framework, SIFT features computation, and matching. Apparently, the processing times are different depending on the size and texture of the object. In our experiments, the number of positive samples was determined by the size of the object, and the number of negative *M* = 200. In addition, *d* = 85, *w* = *h* = 20, *α_1_* = 0.3, *α_2_* = 0.2, *β* = 0.8, and *γ* = 0.8. The particle number for each DUFrag was *M*_P_ = 0.01 × Area (background), and *N*_P_ = 10.

It should be noted that, since each fragment is tracked individually, parallel hardware (such as multi-core processors or Graphics Programmable Units) can be explored to further increase running speed.

### 4.4 Limitations and future works

Even if the tracked object has a large area of homogeneous color, as long as the object is distinct from the background, the proposed algorithm can use the peripheral discriminative fragments for tracking, such as the person in Walking2 sequence. In rare cases where the proposed algorithm may fail, the reasons could be that, all fragments are too homogeneous and do not contain unique fragments, or the object is blurry and does not contain discriminative fragments from the background, or Harris-SIFT tracking failed. Another limitation is that small objects (less than 20 × 20 pixels) are not suitable for fragment-based tracking or fragment selection. How to design more efficient local features, especially for homogeneous and blurry object, as well as small object, is our future work.

More research works are also needed to improve the algorithm’s adaptive capability to severe, non-rigid deformation.

## 5. Conclusion

The conventional features selection usually directly uses the local features selected from the previous frames to locate object while ignoring their validity, and this can easily lead to feature degradation and impair tracking robustness and accuracy. Unlike many existing algorithms, this paper proposes a robust fragment-based object tracking algorithm, which uses discriminative and unique fragments pre-selection mechanism to determine DUFrags, and then uses Harris-SIFT filter and spatial constraints to further identify the current valid fragments (V-DUFrags). This process helps to exclude the occlusion or transformed fragments. Finally, the object is localized using a structured fragment template combining the displacement, structural consistency, and similarity of each valid fragment.

The proposed method of valid fragments determined by SIFT matching and spatial constraint inherits the existing common visual pattern discovery methods. However, the proposed method adopts a simple connection evaluation and structural consistency statistic, instead of the existing strongly connected subgraph method [62], or the factorized graph matching method [63]. Connectivity evaluation allows local deformation of the moving object, and structural consistency ensures that the overall structure of the object does not change very much. Meanwhile, the method is efficient and suitable for real-time object tracking.

Actually, we think that, using valid fragments determined by SIFT matching and spatial constraint, instead of Harris-SIFT directly for tracking, it is possible to use the multiple overlapped candidate fragments, which is coincident with multiple hypotheses of particle filter. In our approach, SIFT deals with Harris corners at a low level, a fragment describes an object at a medium level, and the combination of multiple structured fragments represents the object at a high level. Such a multi-level framework can be more robust than methods that only rely on single level. The experimental results show that the proposed algorithm is accurate and robust in challenging scenarios that include severe occlusions and large-scale changes.

## Figures and Tables

**Table 1:** Summary of notations

Notations	Meaning
DUFrags	Discriminative and unique fragments
V_DUFrags	Valid DUFrags
**T**	Object template
*w*, *h*	The width and length of fragment
*N*	The number of positive samples
*M*	The number of negative samples
$F_{+i}^{t}$	The positive samples
$\Omega_{\mathrm{pos}}=\{F_{+1}^{t},F_{+2}^{t},\ldots ,F_{+N}^{t}\}$	The positive samples set
$F_{-j}^{t}$	The negative samples
$\Omega_{\mathrm{Neg}}=\{F_{-1}^{t},F_{-2}^{t},\ldots ,F_{-M}^{t}\}$	The negative samples set
$D_{i}^{t}$	The discrimination degree between $F_{+i}^{t}$ and *Ω*_Neg_
$U_{i}^{t}$	The maximum difference between the estimated object fragment and other object fragments.
Ω***_DU_*={F_1_, F_2_,** …**, F_NDU_}**	The samples set of DUFrags
*N*_DU_	The samples number of *Ω*_DU_
*M*_P_	The number of particles for each DUFrags.
*N*_P_	The number of top-rank locations using color histogram similarity for each DUFrags.
**Φ**	Candidate fragments for DUFrags
**Φ*_V_DU_***	All the valid fragments in Φ
Ω***_V_DU_***	All the matched valid fragments in Ω_DU_
*τ_1_*, *τ_2_*	The thresholds for DUFrags
*f*_max_, *f*_min_	The maximum and the minimum code frequency for structural consistency
*β, γ*	The thresholds for structural consistency

**Table 2:** Test and verify for fragment size

Sequences	Object size (pixels)	Average center location error (in pixels)					

		No frag	*W* = *h* = 10	*W* = *h* = 15	*W* = *h* = 20	*M* = *h* = 25	*M* = *h* = 30
Faceocc1	80 × 102	10.1	2.8	**2.4**	2.7	3.7	7.5
Sylvester	50 × 61	11.2	61.6	12.5	**10.5**	18.4	19.2
Breadcar	128 × 75	39.2	20.3	15.0	**12.4**	13.2	16.8
Pets	39 × 28	7.7	**5.9**	7.2	8.2	8.2	8.2
Highway	51 × 64	20.1	38.4	18.1	**16.0**	28.8	38.6
Highway2	59 × 57	20.9	19.0	18.5	**18.3**	23.3	21.4
Highway3	41 × 50	14.0	8.3	7.2	**7.2**	9.2	14.9
CAVIAR	52 × 40	7.4	8.7	8.1	7.5	**4.0**	7.1
plane	98 × 64	8.8	4.7	3.11	**2.5**	4.5	4.5
Average	–	15.5	18.9	10.2	9.5	12.6	15.4

The minimum average center location error for eachsequence is displayed in bold

**Table 3:** Score of precision plot (threshold = 20)

Attribute	Proposed SFragT	Staple	KCF	Struck	DFT	LOT	CT
IV	**0.748**	***0.727***	*0.728*	0.558	0.475	0.367	0.359
SV	**0.723**	*0.721*	***0.679***	0.639	0.441	0.465	0.448
OCC	**0.802**	*0.775*	***0.749***	0.564	0.481	0.532	0.412
DEF	*0.779*	**0.788**	***0.740***	0.521	0.537	0.487	0.435
MB	***0.649***	**0.670**	*0.650*	0.551	0.383	0.395	0.306
FM	*0.605*	**0.642**	***0.602***	0.604	0.373	0.420	0.323
IPR	*0.764*	**0.773**	***0.725***	0.617	0.469	0.508	0.356
OPR	*0.756*	**0.764**	***0.729***	0.597	0.497	0.520	0.394
OV	*0.662*	**0.669**	***0.650***	0.539	0.391	0.567	0.336
BC	***0.695***	*0.730*	**0.753**	0.585	0.507	0.529	0.339
LR	*0.488*	**0.505**	***0.381***	0.545	0.211	0.201	0.152
Overall Score	*0.774*	**0.782**	***0.740***	0.656	0.496	0.522	0.406

**Table 4:** Score of success plot (AUC)

Attribute	Proposed SFragT	Staple	KCF	Struck	DFT	LOT	CT
IV	**0.563**	*0.561*	***0.493***	0.428	0.383	0.286	0.295
SV	**0.548**	*0.545*	***0.427***	0.425	0.329	0.335	0.302
OCC	**0.609**	*0.585*	***0.514***	0.413	0.381	0.378	0.321
DEF	*0.604*	**0.607**	***0.534***	0.393	0.439	0.345	0.345
MB	***0.483***	**0.526**	*0.497*	0.433	0.333	0.312	0.269
FM	*0.498*	**0.501**	***0.459***	0.462	0.320	0.331	0.298
IPR	*0.559*	**0.576**	***0.497***	0.444	0.365	0.355	0.282
OPR	*0.547*	**0.569**	***0.495***	0.432	0.387	0.364	0.297
OV	*0.526*	***0.518***	**0.550**	0.459	0.351	0.467	0.359
BC	***0.524***	**0.557**	*0.535*	0.458	0.407	0.385	0.273
LR	*0.358*	**0.395**	***0.312***	0.372	0.200	0.189	0.120
Overall score	*0.585*	**0.593**	***0.514***	0.474	0.389	0.367	0.306

**Table 5:** Processing time

Sequence	Image size (pixel)	Object size (pixel)	Length (frame)	Average processing time (s/f)
FaceOcc1	352 × 288	115 × 163	892	0.6674
Girl	128 × 96	32 × 46	500	0.0767
David3	640 × 480	36 × 132	252	0.3340
Walking2	384 × 288	32 × 116	500	0.1033
Car4	360 × 240	108 × 88	659	0.1567
Sylvester	320 × 240	52 × 62	1345	0.2764
David	320 × 240	65 × 79	471	0.2891
Deer	704 × 400	96 × 66	71	0.0884
